# Distinct sub-MIC kill kinetics of Cu and Ag in *Escherichia coli*

**DOI:** 10.1128/spectrum.03512-25

**Published:** 2026-05-15

**Authors:** Merilin Rosenberg, Sigrit Umerov, Carmen Marianne Teär, Angela Ivask

**Affiliations:** 1 Institute of Molecular and Cell Biology, University of Tartu 117223, Tartu, Estonia; Tel Aviv Sourasky Medical Center, Tel Aviv, Israel; Tenth People's Hospital of Tongji University, Shanghai, China; Ilam University of Medical Sciencesmicrobiology, Ilam, Iran

**Keywords:** tolerance, sub-MIC kill, growth inhibition, MIC, antibacterial, silver, copper, resistance

## Abstract

Copper and silver are well-known and widely used antimicrobial metals that are often considered to employ diverse, overlapping biocidal mechanisms of action, and induce corresponding bacterial defense responses. Exposure to antimicrobial metals at concentrations below the minimal inhibitory concentration (sub-MIC) is widespread in natural, clinical, and built environments, and may influence microbial survival dynamics that could have implications for tolerance or resistance development. By analyzing growth and kill kinetics *of Escherichia coli* under copper or silver exposure, we observed that sub-MIC copper concentrations resulted in a lasting dose-dependent slowing of exponential growth, with reduced yield, while silver seemed to cause dose-dependent growth delay without substantially affecting exponential growth or yield. Time-kill experiments revealed minimal loss of viability in early copper exposure, while in the case of silver, a rapid dose-dependent transient killing followed by normal exponential regrowth of the survivors was observed, underlying the seemingly dose-dependently extended lag phase durations. Distinguishing sustained growth inhibition from transient killing followed by regrowth may be important for interpreting how sub-MIC exposures relate to tolerance and resistance development. Our results suggest that, under these conditions, silver primarily permits regrowth of surviving cells after an initial killing phase, whereas copper imposes sustained growth inhibition. The findings highlight new and known challenges in antimicrobial characterization and risk assessment of metal-based formulations by using widespread non-kinetic endpoint assays such as MIC.

**IMPORTANCE:**

Copper and silver are widely used metals with antimicrobial properties that are often considered to employ diverse, overlapping biocidal mechanisms of action, and corresponding bacterial defense responses. Here, we show that sub-minimal inhibitory concentration (sub-MIC) copper causes lasting, dose-dependent growth inhibition of *Escherichia coli*, while silver seemingly delays otherwise normal growth kinetics. The latter is primarily not caused by growth inhibition, but partial killing by silver, followed by normal regrowth of the survivors. These previously under-recognized differences in sub-MIC toxicity kinetics suggest that copper and silver create distinct short-term survival and growth regimes, which may have implications for future studies of tolerance and resistance development. Under the conditions tested, silver-associated growth delay was explained by survival and regrowth of a reduced subpopulation, whereas copper exposure required continued growth under inhibitory conditions. This difference imposes new challenges for the design and application-relevant risk assessment of metal-based antimicrobial formulations.

## OBSERVATION

Silver and copper are widely used in various antimicrobial applications with the intent to kill or limit the spread of potentially pathogenic microbes, yet their mechanisms of action are not fully understood. Cellular targets of the antibacterial activity of copper and silver, as well as the corresponding bacterial defenses, are often considered overlapping due to similar coordination chemistry and sulfhydryl reactivity, leading to shared efflux and sequestration mechanisms (1–5). For example, the CusCFBA system exports both Cu(I) and Ag(I) and is induced by CusRS (5). Unlike toxic silver, copper is an essential micronutrient tightly regulated by homeostasis but becomes toxic in excess (1, 6). A key difference between copper and silver is Cu(I)/Cu(II) redox activity, which generates reactive oxygen species and causes direct damage such as lipid peroxidation (7). We have previously found that silver and copper also differ in their relative toxicity to bacteria depending on exposure conditions. *Escherichia coli* tolerates higher copper ion concentrations (millimolar range) than silver ion concentrations (micromolar range) in liquid exposure media but survives longer on silver surfaces than on copper surfaces in semi-dry exposure conditions (8, 9). These contrasts suggest distinct antibacterial mechanisms or condition-dependent defense responses.

Antibacterial activity is typically measured and compared via static endpoints, not kinetic assays. Minimal inhibitory concentration (MIC) indicates the presence or absence of growth after standardized exposure, while minimal biocidal concentration only reflects endpoint mortality (>99.9%) (10, 11). However, endpoint assays can mask regrowth after transient killing. To capture dynamics, where an antimicrobial agent is quickly detoxified and/or causes transient killing that allows survivors to regrow before the assay endpoint, growth and kill kinetics assays in well-defined experimental conditions should be used. Implications of the strain, media, and exposure conditions selection in the current study are further discussed in Supplemental note 1.

**Fig 1:**
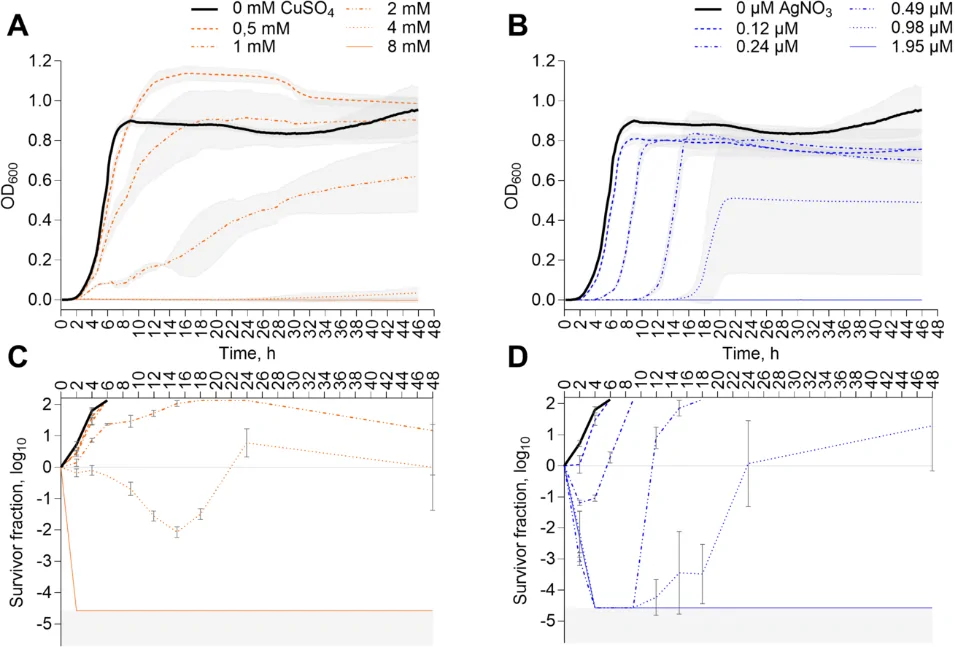
Growth (**A and B**) and kill (**C and D**) kinetics of *Escherichia coli* BW25113 exposed to CuSO_4_ (**A and C**) or AgNO_3_ (**B and D**) in the range of 0.0625–1× an operational MIC value defined based on absence of detectable growth over 48 h. MIC values corresponding to standard EUCAST/CLSI timepoints (16–20 h) and the operational 48 h endpoint were 4 mM and 8 mM for Cu, respectively, and 1.95 µM for Ag at both timepoints. Mean and SD of three (**C and D**) to four (**A and B**) biological replicates are shown. All growth curves from individual experiments represented in panels **A** and **B** can be found in Fig. S3, and survival data in panels **C** and **D** are presented as bar graphs on Fig. S5, correlation between metal concentration and growth parameters in Fig. S2, and numerical data in File S1. Gray shading in panels **A** and **B** denotes standard deviation, and in panels **C** and **D**, the value range below the post-exposure colony counting detection limit, with no colonies detected.

Silver and copper appear to affect *E. coli* growth kinetics differently. To study the effects of copper and silver on the growth and viability of *E. coli*, exponential cultures were exposed to 0.0625 to 1 times the 48 h MIC value increments of the respective metal salts in MOPS-buffered defined medium (Fig. 1; detailed methods of the growth and time-kill experiments presented can be found in Supplementary Note 2). At comparable sub-MIC concentration ranges, CuSO₄ increased doubling time and reduced biomass yield of *E. coli* in a dose-dependent manner (Fig. 1A; Fig. S1 and S2). In contrast, AgNO₃ seemingly prolonged lag time with little effect on doubling time or yield (Fig. 1B; Fig. S1 and S2).

Silver and copper cause distinctly different sub-MIC kill kinetics during the ostensible lag phase. Copper exposure resulted in minimal early killing in addition to lasting growth inhibition (Fig. 1A and C), whereas silver caused rapid, dose-dependent, up to over 4-log reduction in viable counts prior to resumption of normal exponential growth (Fig. 1B and D). Prolonged physiological lag time can signal a stress response (12, 13) and, without the time-kill data in Fig. 1D, could plausibly account for the delayed growth in Fig. 1B. However, a decrease in inoculum density also increases population-level lag phase duration—a phenomenon commonly known as the inoculum effect (12, 14). The latter was also evident for *E. coli* under our growth-assay conditions (Fig. S1 and S4). Although much higher temporal resolution in early exposure would be needed to calculate and compare kill rates across concentrations, a rapid, up to >4-log killing followed by regrowth displaying dose-dependently increased lag phase duration under antimicrobial exposures, as in the case of silver in Fig. 1D and B, could be primarily attributable to sub-MIC killing and reduced viable cell count at resumption of growth. In case of observed lag shifts, growth kinetics should be complemented by sub-MIC tolerance assays to discriminate partial killing from physiologically prolonged lag phase.

Although the present observations were obtained in a single strain under defined conditions and therefore represent a controlled proof-of-principle rather than a generalizable assessment across taxa, they reveal distinct short-term phenotypic responses to sub-MIC copper and silver exposure. Under the conditions tested, silver caused transient killing followed by regrowth of survivors, whereas copper caused sustained growth inhibition with minimal early killing. These phenotypic differences now warrant future mechanistic studies of metal uptake, intracellular speciation, damage, and defense responses. The different survival and growth dynamics may also be relevant to how repeated sub-MIC metal exposures shape tolerance- or resistance-associated trajectories, but such long-term outcomes were not tested here and will require dedicated evolution and competition experiments. Tolerance—survival without growth—is easier to develop than resistance, which requires enhanced growth in the presence of an antimicrobial agent. Tolerance is thus considered a precursor to resistance (15, 16), offering survivors time to acquire adaptive traits. While both silver and copper are used in commercial antimicrobial products (e.g., wound care, textiles, touch surface materials), copper resistance is more studied in the context of environmental pollution or colonization of the indoor environment. On the other hand, silver resistance is a well-known challenge necessitating combined treatment strategies, for example, in burn wound care (17, 18). These observations indicate that the short-term phenotypic requirements for survival under silver and copper exposure differ: under the tested silver conditions, regrowth depended on survival of an initially reduced subpopulation, whereas under copper exposure, continued growth occurred under sustained inhibition. The findings raise the possibility that silver- and copper-based antimicrobial applications may differ in how they relate to antibiotic tolerance and resistance outcomes under repeated exposure. In conclusion, our observations demonstrate that endpoint assays such as MIC can obscure distinct sub-MIC toxicity kinetics, which may be relevant for interpreting antimicrobial activity and guiding future risk assessment frameworks.

## Supplementary Material

Reviewer comments

## SUPPLEMENTAL MATERIAL

File S1 spectrum.03512-25-s0001.xlsx Supplemental data file.

Supplemental materials spectrum.03512-25-s0002.pdf Supplemental notes and Figures S1 to S5.

OPEN PEER REVIEW reviewer-comments.pdf An accounting of the reviewer comments and feedback.

ASM does not own the copyrights to Supplemental Material that may be linked to, or accessed through, an article. The authors have granted ASM a non-exclusive, world-wide license to publish the Supplemental Material files. Please contact the corresponding author directly for reuse.
