# Supplementary material for: Distinct sub-MIC kill kinetics of Cu and Ag in Escherichia coli
Source: Microbiol Spectr. 2026 May 15;14(7):e03512-25. doi: 10.1128/spectrum.03512-25 (PMC13340094; doi:10.1128/spectrum.03512-25)
Supplement: Supplemental materials — Supplemental notes and Figures S1 to S5. [file spectrum.03512-25-s0002.pdf]

Supplementary Notes and Figures for:

Distinct sub-MIC kill kinetics of Cu and Ag in *Escherichia coli*

Merilin Rosenberg, Sigrit Umerov, Carmen Marianne Teär, Angela Ivask

Institute of Molecular and Cell Biology, University of Tartu, Riia 23, 51010 Tartu, Estonia

Corresponding author: Merilin Rosenberg; merilin.rosenberg@ut.ee

Supplementary Note 1 on strain selection, growth and exposure conditions.

Strain and inoculum selection

We first observed the effects described in this study for *E. coli* ATCC 8739 (unpublished data), a strain that is routinely used in antimicrobial testing. However, the strain tends to rapidly acquire silver resistance in liquid medium granted by mutations in the cryptic genomic *sil* locus (1–4) that interferes with studying the effects of antimicrobial metals on the wild-type strain. *E. coli* K-12 derivative BW25113 (5) was used for the current study as a better described model strain of *E. coli* lacking the *sil* locus. Exponential phase inoculum was used in the study similarly to standardized MIC and MBC assays and to avoid potential biases caused by inoculating stationary or biofilm cultures with different tolerance profiles into fresh growth-supporting exposure medium. Due to the limited growth phase coverage, the results cannot be generalized to stationary and colony-biofilm cultures in which cases additional experiments would be necessary.

Exposure medium selection

In complex environments such as biofilms or organic-rich matrices, metal speciation and microbial interactions may substantially alter the observed kinetics. However, proof-of-principle research must first study biological effects in controlled conditions with minimal confounding factors. In addition to strain and growth phase selection, the growth medium to our best knowledge least iterfering the metal-microbe interactions was selected as described below.

It has been demonstrated before that transferring *E. coli* from pH=7.0 to pH=4.2 medium can substantially decrease viable counts (6) and pH=5.5 also seems to both decrease growth rate and prolong lag phase at population level compared to pH=7.5 (7). In addition, during exponential growth phase *E. coli* culture itself can further decrease pH of a glucose-supplemented medium by 1.5-2 pH units (8, 9).

In the context of antimicrobial metals, the addition of 365 ppm (5.7 mM) or 650 ppm (10 mM) copper to regular unbuffered medium such as LB has been shown to decreases pH of the medium from 7.2 to 5.3 or 4.7 accordingly (10), an acidity range that impairs growth of *E. coli* also without excess copper. Similarly, also in our experience supplementing LB (Lennox salt) with $CuSO_4$ reduces pH while the pH change is expected to be much smaller in the buffered MOPS Minimal Medium (MMM), figure **to the left** ←, whereas silver had no effect on pH of the media (data not shown).

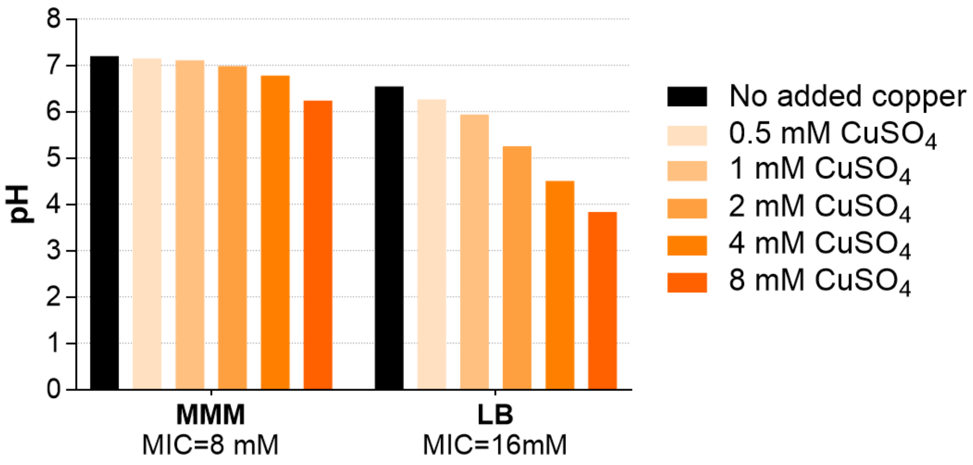

Additionally, acid resistance, pH recovery and growth dynamics of *E. coli* subjected to near-lethal acid stress in unbuffered LB also depends on inoculum density with complete growth inhibition at initial pH≤4.0 and MIC assay cell density while increasing pH to above ~4.4 is needed before resuming growth (11). This further complicates interpretation of results with varying cell counts or potentially partial initial killing by either acidity or copper or both. Additional toxic effect of acidification due to copper in the medium might spark ideas for combined synergistic use of antimicrobial agents. However, the slow-release copper sources such as CuO nanoparticles do not seem to have a similar effect on medium pH.

MMM (12, 13) was selected for metal exposures due to its buffering capacity to reduce pH bias while comparing antimicrobial activity of copper and silver. Buffered medium counteracts acidification upon addition of $CuSO_4$ and helps to maintain the pH near the optimal growth range of *E. coli* (pH ~6.5-7.5) at sub-MIC copper concentrations. Metal ion toxicity is heavily affected by exposure medium (14, 15). MMM was also used due to its defined characteristics and lower organic content to control the metal ion speciation effects on bioavailability and thus toxicity to bacteria (*e.g.* formation of insoluble salts and complexing of metal ions by thiol groups). Both silver and copper ions can be complexed by organic contents, especially cysteine residues, of the medium with variable affinity, while only silver forms practically insoluble AgCl in the presence of ~60 mM chloride ions in MMM. However, similar situations would be encountered in clinically relevant conditions. Chloride content of MMM is lower than in LB (Miller 10 g/L NaCl → 171 mM; Lennox 5g /L NaCl → 86 mM), PBS (140 mM) or human blood plasma (~100 mM).

Re-growth conditions

We have previously observed that copper exposure can cause slower colony growth substantially affecting results of agar MIC assays (2) possibly also due to copper-induced pH changes. Therefore, growth and kill kinetics were recorded for 48 h instead

of 24 h. Drop-plated colonies from the kill curve experiments were counted after 14-16 h for optimal countable colony size and re-checked after 24, 48 and 72 h. Spread-plates from kill curves were counted after 24 h and checked after 48 and 72 h for possible slower growth from exposures with higher metal concentration. No delayed emergence of colonies was observed. 96-well exposure plates from the growth and kill kinetics experiments were kept at 37°C and 150 rpm for 5 days after the 48-h data collection and visually confirmed that delayed growth (emergence of visible turbidity) was not observed for metal concentrations at which no OD increase had been detected during 48 h.

Supplementary Note 2 on method details

Escherichia coli K-12 derivative BW25113 was used for the current study as a well-described model strain of *E. coli*. Lysogeny broth (LB: 5 g/L yeast extract, 10 g/L tryptone, 5 g/L NaCl with optional 15 g/L bacteriological agar) and 37°C incubation with or without 150 rpm orbital shaking was used for all precultures and phosphate-buffered saline (PBS: 8 g/L NaCl, 0.2 g/L KCl, 1.44 g/L Na_2HPO_4, 0.2 g/L KH_2PO_4; pH 7.1) for washing and serial dilutions of cultures. Metal exposures used MOPS Minimal Medium (MMM)(12, 13) supplemented with 0.4% glucose, 0.4% casamino acids, and 20 µg/L tryptophan (~60 mM chloride in 1× MMM as described above).

Second subculture of *E. coli* on LB was used to inoculate overnight liquid LB cultures. The stationary culture was 100-fold diluted into fresh LB and grown to exponential phase (OD_{600}=0.5-0.7), then washed twice with cold PBS (4°C, 4400 g, 10 min). Pellets were resuspended in 2× MMM and adjusted to OD_{600} = 0.002 (~10^6 CFU/mL). Inoculum was added to twofold dilution series of $AgNO_3$ or $CuSO_4$ or mixed 1:1 with deionized water and serially diluted in 1× MMM to test inoculum size effects. The final volume of exposures in 96-well suspension culture plates was 150 µl for growth curves and 200 µl for kill curves. Sterile water was pipetted between the wells and plates sealed with parafilm to reduce drying during long incubations.

Growth curves were measured at OD_{600} every 15 min at 37°C with double-orbital mixing (BioTek Synergy H1, Agilent). Blank-corrected data were analyzed with Dashing Growth Curves at https://dashing-growth-curves.ethz.ch/ (16).

Minimal inhibitory concentrations (MIC) of $AgNO_3$ or $CuSO_4$ were determined from growth curves as the lowest concentration at which no time-dependent OD increase was registered during the experiments and verified by no visible growth after 48 h incubation. MIC values of 4 mM and 8 mM Cu after 18 and 48 h, respectively, or 1.95 µM Ag after both 18 h and 48 h are stated in the results to represent 16-20 h standard condition for MIC determination as well as operational definitions aligned with the experimental design of the study.

For kill curves, exposure plates were incubated at 37°C, 150 rpm in the dark. At each time-point, three concentration series per metal were serially diluted in PBS and drop-plated on LB; 150 µL of undiluted exposures were also spread-plated on 10 cm LB plates for viable counts. Survival was expressed as log_{10}-transformed survivor fraction (post-/pre-exposure viable counts).

Statistical analysis was done in GraphPad Prism 10.4.1 using correlation, linear regression and one-way ANOVA with post-hoc testing for multiple comparisons at α = 0.05 across 3–4 biological replicates, as detailed in figure legends.

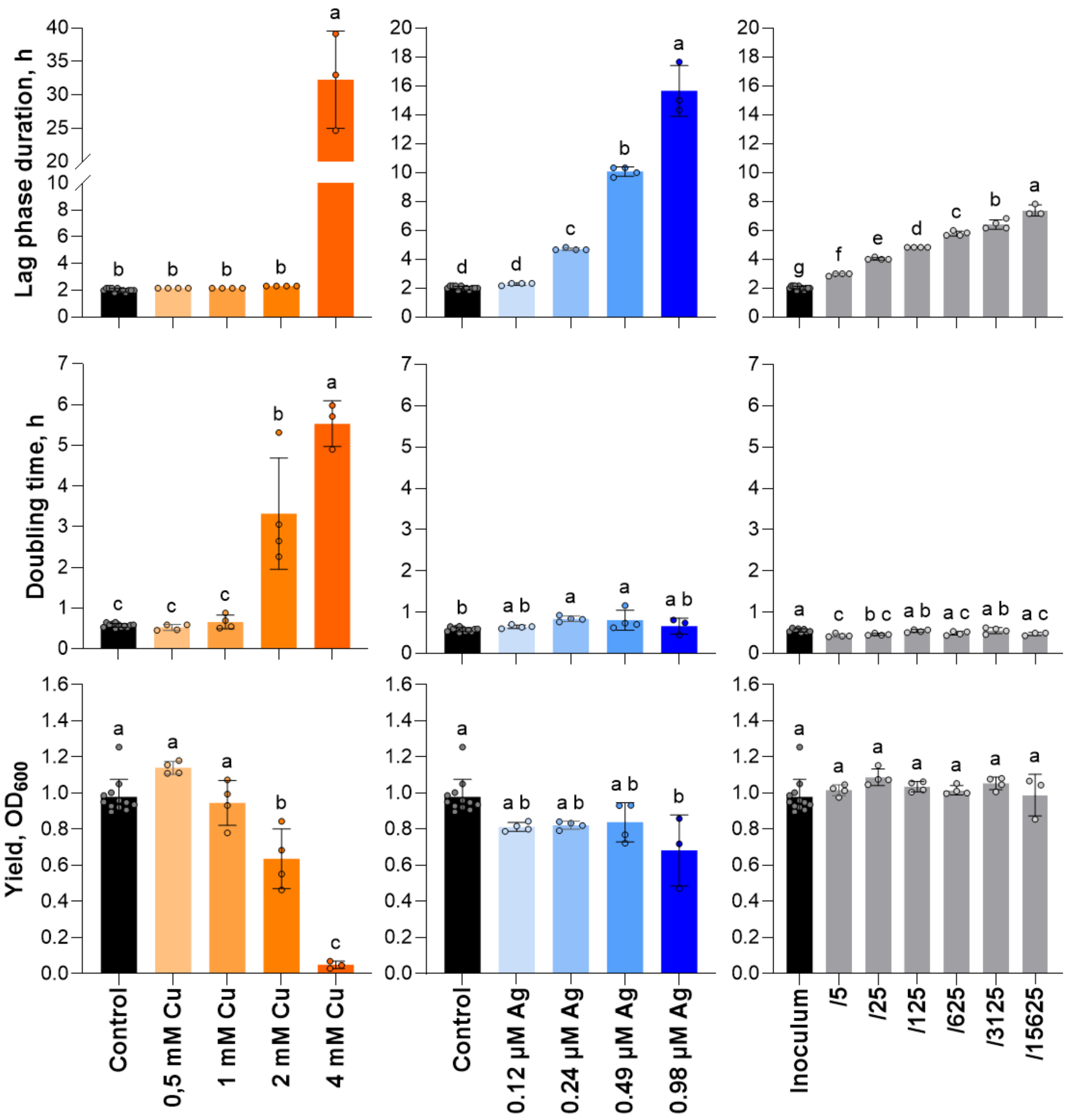

Supplementary Figure S1. Growth parameters of *E. coli* BW25113 exposed to $CuSO_4$ (orange) or $AgNO_3$ (blue) or inoculums with different viable cell densities (grey). Values are calculated from growth curves on Supplementary Figures S3 and S4. Nominal metal concentration or inoculum fold dilution is marked on X-axis where appropriate. Single data points with mean and SD are shown. Lower case letters denote similarity groups based on one-way ANOVA followed by Tukey post-hoc test for multiple comparisons at $\alpha=0.05$.

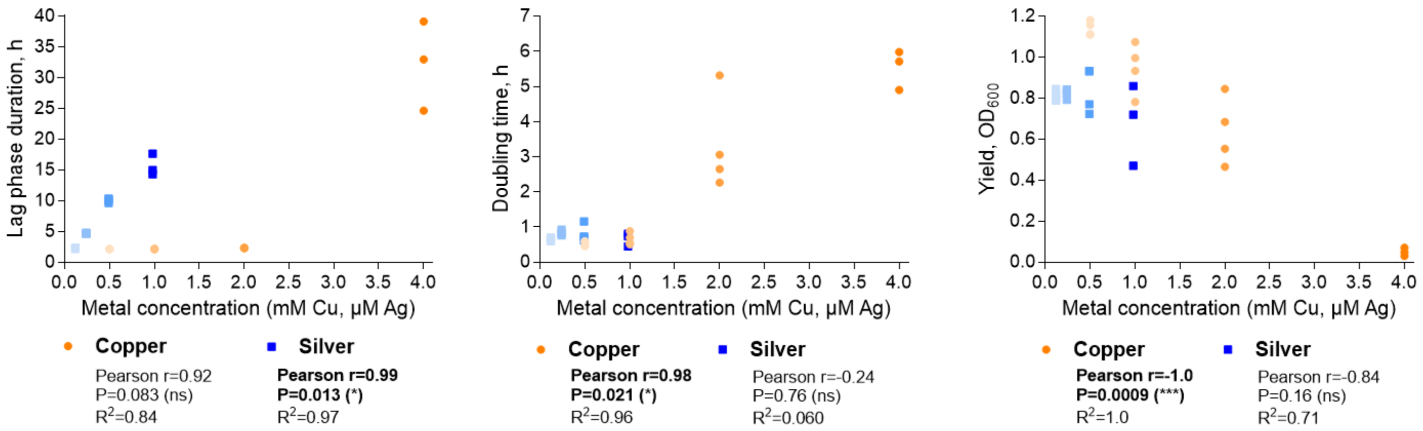

Supplementary Figure S2. Dose-dependent changes in lag phase duration (left), doubling time (middle) and yield (right) in response to sub-MIC copper (orange gradient) or silver (blue gradient) concentration. Statistically significant linear correlations are marked in bold. Growth parameter values calculated from growth curves on Supplementary Figure S3 and presented on Supplementary Figure S1 were used for correlations. Results from 4 biological replicates are presented.

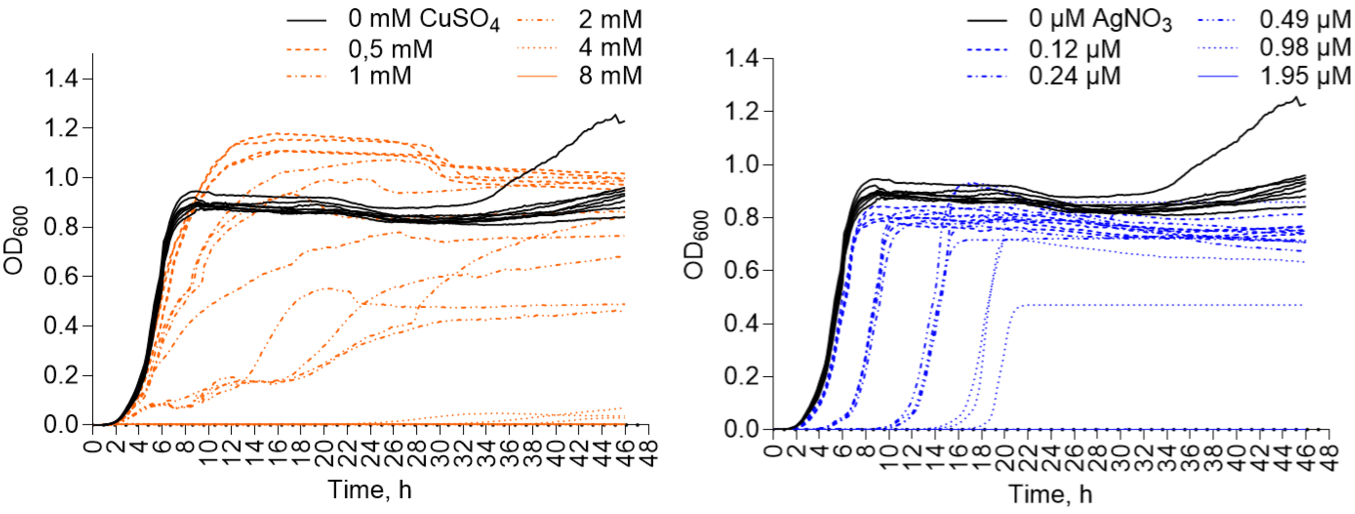

Supplementary Figure S3. Growth curves of *E. coli* BW25113 in the presence or absence of $CuSO_4$ (left, orange) or $AgNO_3$ (right, blue). Individual curves from 4 experiments underlying the mean curves on Figure 1 are shown. Non-exposed controls (black) on both panels are pooled across all experiments.

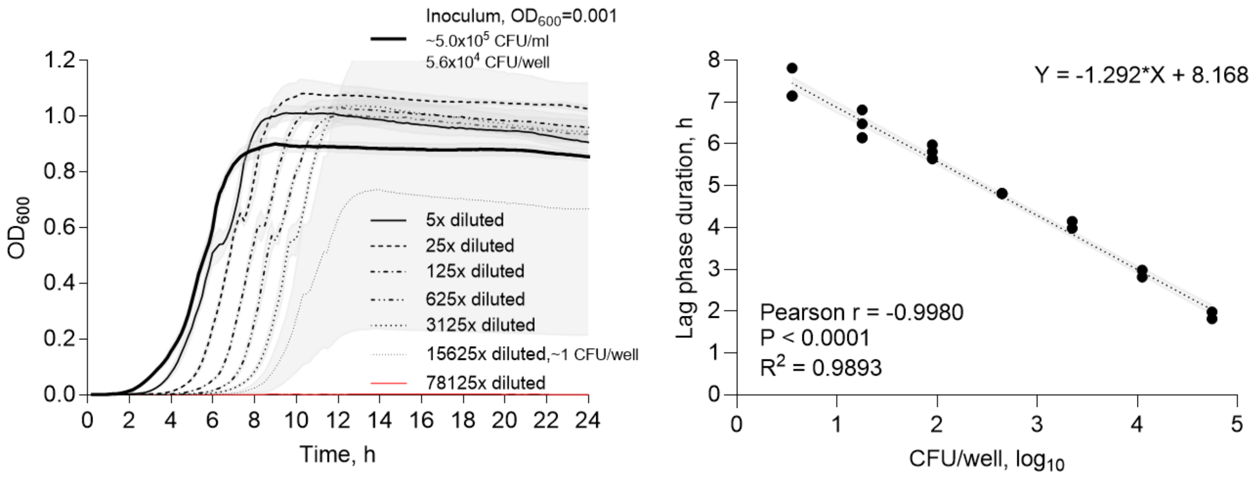

Supplementary Figure S4. Effect of inoculum density of *E. coli* BW25113 on growth curves (left) and observed strong negative correlation between the lag phase duration and inoculum density (right). Dotted line denotes linear regression. Results from 4 biological replicates are presented.

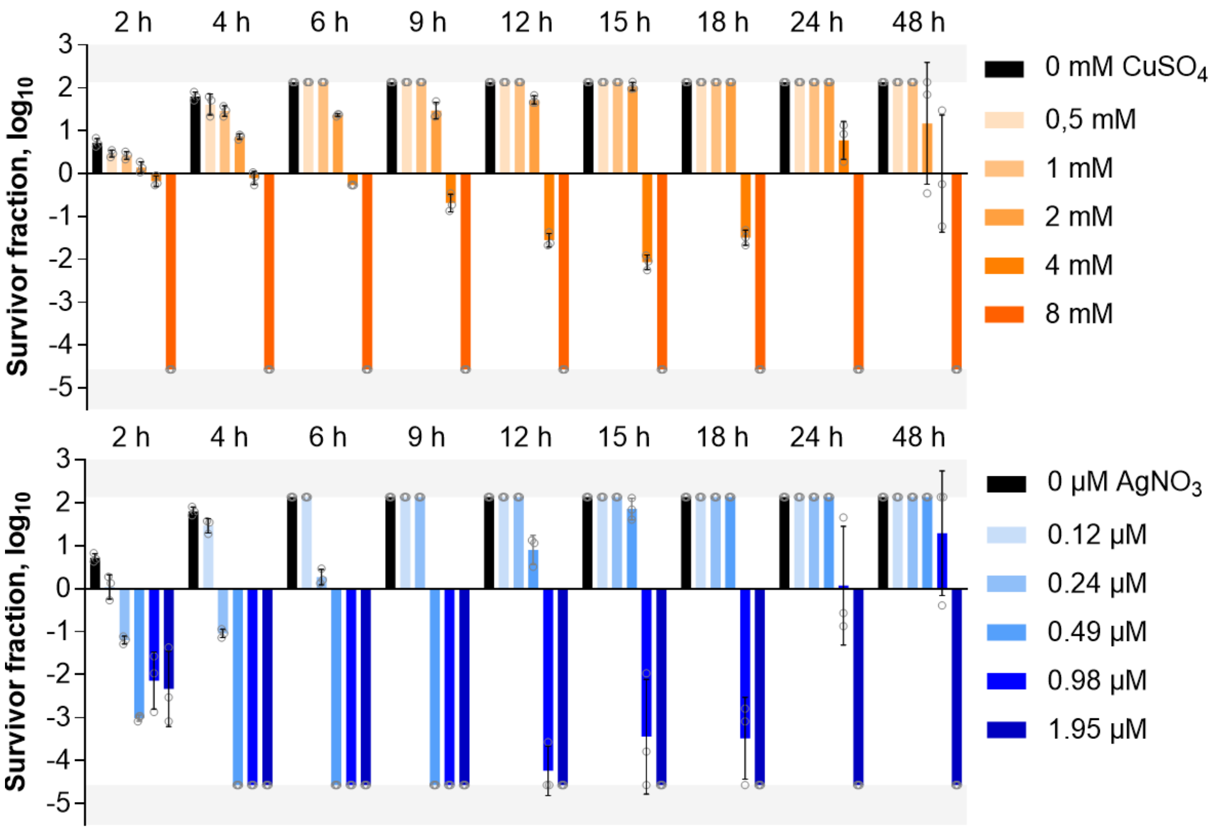

Supplementary Figure S5. Survival of *Escherichia coli* BW25113 (mean 5.74 log_{10}CFU/ml) exposed to $CuSO_4$ (orange, upper panel) or $AgNO_3$ (blue, lower panel) during 48 h exposure. Mean and SD of 3 experiments underlying the kill curves on Figure 1 are shown. Areas highlighted in grey denote ranges exceeding colony counting detection limits.

References for Supplementary Notes and Figures

1. Randall CP, Gupta A, Jackson N, Busse D, O'Neill AJ. 2015. Silver resistance in Gram-negative bacteria: a dissection of endogenous and exogenous mechanisms. J Antimicrob Chemother 70:1037–1046.

2. Rosenberg M, Park S, Umerov S, Ivask A. 2025. Experimental evolution of *Escherichia coli* on semi-dry silver, copper, stainless steel, and glass surfaces. Microbiol Spectr 13:e02173-24.

3. Woolley CA, Sutton JM, Wand ME. 2022. Mutations in SilS and CusS/OmpC represent different routes to achieve high level silver ion tolerance in Klebsiella pneumoniae. BMC Microbiol 22:113.

4. Blanco Massani M, Klumpp J, Widmer M, Speck C, Nisple M, Lehmann R, Schuppler M. 2018. Chromosomal Sil system contributes to silver resistance in E. coli ATCC 8739. BioMetals 31:1101–1114.

5. Baba T, Ara T, Hasegawa M, Takai Y, Okumura Y, Baba M, Datsenko KA, Tomita M, Wanner BL, Mori H. 2006. Construction of *Escherichia coli* K-12 in-frame, single-gene knockout mutants: the Keio collection. Mol Syst Biol 2:2006.0008.

6. Xu Y, Zhao Z, Tong W, Ding Y, Liu B, Shi Y, Wang J, Sun S, Liu M, Wang Y, Qi Q, Xian M, Zhao G. 2020. An acid-tolerance response system protecting exponentially growing Escherichia coli. Nat Commun 11:1496.

7. Zhang W, Chen X, Sun W, Nie T, Quanquin N, Sun Y. 2020. Escherichia coli Increases its ATP Concentration in Weakly Acidic Environments Principally through the Glycolytic Pathway. Genes 11:991.

8. Sánchez-Clemente R, Igeño MI, Población AG, Guijo MI, Merchán F, Blasco R. 2018. Study of pH Changes in Media during Bacterial Growth of Several Environmental Strains, p. 1297. *In* Environment, Green Technology, and Engineering International Conference. MDPI.

9. Vivijs B, Moons P, Aertsen A, Michiels CW. 2014. Acetoin Synthesis Acquisition Favors Escherichia coli Growth at Low pH. Appl Environ Microbiol 80:6054–6061.

10. Gunawan C, Teoh WY, Marquis CP, Amal R. 2011. Cytotoxic Origin of Copper(II) Oxide Nanoparticles: Comparative Studies with Micron-Sized Particles, Leachate, and Metal Salts. ACS Nano 5:7214–7225.

11. Segura Munoz RR, Sourjik V. 2025. Collective dynamics of *Escherichia coli* growth under near-lethal acid stress. mBio e01932-25.

12. Neidhardt FC, Bloch PL, Smith DF. 1974. Culture Medium for Enterobacteria. J Bacteriol 119:736–747.

13. MOPS Minimal Medium. E. coli Genome Project at the University of Wisconsin-Madison.

14. Levard C, Mitra S, Yang T, Jew AD, Badireddy AR, Lowry GV, Brown GE. 2013. Effect of Chloride on the Dissolution Rate of Silver Nanoparticles and Toxicity to *E. coli*. Environ Sci Technol 47:5738–5745.

15. Rewak-Soroczynska J, Dorotkiewicz-Jach A, Drulis-Kawa Z, Wiglusz RJ. 2022. Culture Media Composition Influences the Antibacterial Effect of Silver, Cupric, and Zinc Ions against Pseudomonas aeruginosa. Biomolecules 12:963.

16. Reiter MA, Vorholt JA. 2024. Dashing Growth Curves: a web application for rapid and interactive analysis of microbial growth curves. BMC Bioinformatics 25:67.
