# Supplementary material for: Distinct sub-MIC kill kinetics of Cu and Ag in Escherichia coli
Source: Microbiol Spectr. 2026 May 15;14(7):e03512-25. doi: 10.1128/spectrum.03512-25 (PMC13340094; doi:10.1128/spectrum.03512-25)
Supplement: Reviewer comments [file reviewer-comments.pdf]

AMERICAN SOCIETY FOR MICROBIOLOGY | Microbiology Spectrum

Distinct sub-MIC kill kinetics of Cu and Ag in *Escherichia coli*

Merilin Rosenberg, Sigrit Umerov, Carmen Teär, and Angela Ivask

Corresponding Author(s): Merilin Rosenberg, Tartu Ulikool

Review Timeline:

Submission Date:	November 5, 2025
Editorial Decision:	January 6, 2026
Revision Received:	January 29, 2026
Editorial Decision:	February 22, 2026
Revision Received:	April 10, 2026
Accepted:	April 19, 2026

Editor: Mor Lurie-Weinberger

Reviewer(s): Disclosure of reviewer identity is with reference to reviewer comments included in decision letter(s). The following individuals involved in review of your submission have agreed to reveal their identity: Feng Yang (Reviewer #3); Behrooz Sadeghi Kalani (Reviewer #4)

Transaction Report:

(Note: With the exception of the correction of typographical or spelling errors that could be a source of ambiguity, letters and reports are not edited. The original formatting of letters and referee reports may not be reflected in this compilation.)

DOI: https://doi.org/10.1128/spectrum.03512-25

Re: Spectrum03512-25 (Distinct sub-MIC kill kinetics of Cu and Ag in *Escherichia coli*)

Dear Dr. Merilin Rosenberg:

Thank you for the privilege of reviewing your work. Below you will find my comments, instructions from the Spectrum editorial office, and the reviewer comments.

Please return the manuscript within 60 days; if you cannot complete the modification within this time period, please contact me. If you do not wish to modify the manuscript and prefer to submit it to another journal, notify me immediately so that the manuscript may be formally withdrawn from consideration by Spectrum.

Revision Guidelines
To submit your modified manuscript, log into the submission site at https://spectrum.msubmit.net/cgi-bin/main.plex. Go to Author Tasks and click the appropriate manuscript title to begin. The information you entered when you first submitted the paper will be displayed; update this as necessary. Note the following requirements:

• Upload point-by-point responses to the issues raised by the reviewers in a file named "Response to Reviewers," NOT in your cover letter.
• Upload a compare copy of the manuscript (without figures) as a "Marked-Up Manuscript" file.
• Upload a clean .DOC/.DOCX version of the revised manuscript and remove the previous version.
• Each figure must be uploaded as a separate, editable, high-resolution file (TIFF or EPS preferred), and any multipanel figures must be assembled into one file.
• Any supplemental material intended for posting by ASM should be uploaded with their legends separate from the main manuscript. You can combine all supplemental material into one file (preferred) or split it into a maximum of 10 files with all associated legends included.

For complete guidelines on revision requirements, see our Submission and Review Process webpage. Submission of a paper that does not conform to guidelines may delay acceptance of your manuscript.

Data availability: ASM policy requires that data be available to the public upon online posting of the article, so please verify all links to sequence records, if present, and make sure that each number retrieves the full record of the data. If a new accession number is not linked or a link is broken, provide Spectrum production staff with the correct URL for the record. If the accession numbers for new data are not publicly accessible before the expected online posting of the article, publication may be delayed; please contact production staff (Spectrum@asmusa.org) immediately with the expected release date.

Publication Fees: For information on publication fees and which article types are subject to charges, visit our website. If your manuscript is accepted for publication and any fees apply, you will be contacted separately about payment during the production process; please follow the instructions in that e-mail. Arrangements for payment must be made before your article is published.

ASM Membership: Corresponding authors may join or renew ASM membership to obtain discounts on publication fees. Need to upgrade your membership level? Please contact Customer Service at Service@asmusa.org.

The ASM Journals program strives for constant improvement in our submission and publication process. Please tell us how we can improve your experience by taking this quick Author Survey.

Thank you for submitting your paper to Spectrum.

Sincerely,
Mor Lurie-Weinberger
Editor
Microbiology Spectrum

Reviewer #1 (Comments for the Author):

The manuscript addresses an important and timely issue: the limitations of endpoint MIC-based assays for evaluating antimicrobial effects of metals such as copper and silver. The study highlights how sub-MIC exposures can produce complex growth and killing dynamics that may be obscured by standard assays. The work is clearly written, the experimental logic is sound, and the question is relevant to antimicrobial stewardship and product evaluation.
However, while the findings are interesting, the current version of the manuscript has several limitations in scope, experimental

depth, and evidentiary support that, taken together, may prevent acceptance as a full research article in its present form. Below, I outline specific concerns that would need to be addressed.
The study is based on a single laboratory strain (Escherichia coli BW25113). Given the known strain-specific variability in metal tolerance and resistance mechanisms, the generality of the conclusions is uncertain. While the discussion acknowledges uncertainty in copper and silver toxicity mechanisms, no experiments are included to test specific hypotheses (e.g., oxidative stress, membrane damage, metal uptake or efflux).
The conclusion that silver-induced lag reflects transient killing followed by regrowth is plausible, but would benefit from complementary assays such as viability staining, single-cell analyses, or persister/tolerant fraction measurements. MIC values are central to the framing of "sub-MIC" exposure, yet the manuscript does not clearly detail how MICs were determined under the same experimental conditions or whether MICs remain stable over time.
Toxicity of Cu and Ag depends strongly on ion speciation and chemical state. Without measurements or controls for free ion activity or complexation, reproducibility and cross-study comparability are uncertain. Also, time-kill methodology lacks some critical details
Growth and killing trends are primarily interpreted visually. Explicit statistical modeling of growth rates, lag times, killing rates, and effect sizes would strengthen the conclusions.
Claims regarding antimicrobial testing practices and risk assessment would be stronger if supported by at least one additional strain or species.
The manuscript draws broad implications for antimicrobial product evaluation and resistance development that may not be fully supported by the relatively compact dataset presented.
While the manuscript raises an important conceptual point and presents solid preliminary data, its current form may not meet the evidentiary and scope expectations for a full research article. Substantial revision-particularly expanding experimental breadth, strengthening mechanistic or evolutionary support, and tightening the discussion-would be required.

Reviewer #2 (Comments for the Author):

In this manuscript Rosenberg, et al examine growth behavior of E. coli in the presence of sub inhibitory concentrations of silver and copper, two well-known metals with antimicrobial properties. Overall the manuscript was well written and provided an interesting data set, described and discussed well by the authors. Included below are a few comments that the authors may wish to address to further strengthen the manuscript and add clarity for readers.
1. Figure one C/D was more difficult to interpret the significant of the range "whiskers". Not sure if some spots they were overlapping, but I struggled with them particularly at the right side of the two plots.
2. Remove e.g. in line 110 or restructure the sentence.
3. I think the conclusion line could use further argument/discussion for justification or should be more directly correlated with the data in this observation manuscript.

Thank you for the opportunity to review.

Response to the reviewers

We thank the reviewers for their careful evaluation of our manuscript and for the constructive comments provided. We would like to emphasize that this study was intentionally submitted as an *Observation* (short description of research results of interest to the broad microbiology community: maximum 1,200 words with a maximum of 2 figures and 25 references) and not a full Research Article, to report a focused and potentially impactful kinetic observation rather than to provide a comprehensive mechanistic analysis. Accordingly, the scope of the work is limited to a single well-defined experimental system and a compact dataset designed to highlight distinct sub-MIC growth and kill kinetics of copper and silver that are not captured by widely used endpoint assays.

Below, reviewer comments (in **black**) are addressed one-by-one by the authors (in **blue**) with cited text indented and in *Italic*. All cited references are listed at the end of the document.

Reviewer #1 (Comments for the Author):

The manuscript addresses an important and timely issue: the limitations of endpoint MIC-based assays for evaluating antimicrobial effects of metals such as copper and silver. The study highlights how sub-MIC exposures can produce complex growth and killing dynamics that may be obscured by standard assays. The work is clearly written, the experimental logic is sound, and the question is relevant to antimicrobial stewardship and product evaluation. However, while the findings are interesting, the current version of the manuscript has several limitations in scope, experimental depth, and evidentiary support that, taken together, may prevent acceptance as a full research article in its present form.

Below, I outline specific concerns that would need to be addressed.
The study is based on a single laboratory strain (Escherichia coli BW25113). Given the known strain-specific variability in metal tolerance and resistance mechanisms, the generality of the conclusions is uncertain.

> We agree that metal tolerance and resistance can vary across *E. coli* strains, growth phases, physiological states, and between species. These limitations, including the choice of strain and growth phase, are explicitly acknowledged and discussed in Supplementary Note 1 (section *“Strain and inoculum selection”).*
>
> The kinetic effects reported here were initially observed in *Escherichia coli* ATCC 8739 strain. However, this strain rapidly acquires reduced silver and copper susceptibility due to stochastic mutations in the chromosomal *sil* locus, as demonstrated previously by us and others (Blanco Massani et al., 2018; Rosenberg et al., 2025). This instability precludes its use for robust quantitative analyses. We therefore selected the stable K-12 derivative BW25113 as a model strain, which also enables potential future

functional follow-up using established genetic resources such as the KEIO mutant collection.

While the discussion acknowledges uncertainty in copper and silver toxicity mechanisms, no experiments are included to test specific hypotheses (e.g., oxidative stress, membrane damage, metal uptake or efflux).

We acknowledge that a broad and partially overlapping arsenal of toxicity and defense mechanisms has been described for both copper and silver (main text, lines 46–55). Unlike most antibiotics, metals exert multimechanistic effects, and more often than not the question is not what happened as a population-level sum of events during the exposure but rather what ultimately caused the irreversible changes or overwhelmed the bacterial defenses that eventually lead to cell death and what is collateral or even post-mortem damage. Consequently, detection of *e.g.* membrane damage, envelope stress, loss of proton motive force, DNA damage, or oxidative stress responses does not establish any single mechanism as the primary cause of cell death.

In the context of the present study, population-level mechanistic assays would also be poorly suited to address the key observation in Fig. 1D, where survival of a minute subpopulation following transient silver-induced killing determines the apparent growth delay. Such assays would primarily describe the majority of cells that did not survive the exposure rather than the properties enabling regrowth. Nevertheless, the kinetic data allow one mechanistic inference: copper causes sustained growth inhibition (Fig 1A), whereas the measurable antibacterial effect of silver is transient (Fig 1B). Together with prior evidence for exposure-condition–dependent toxicity of these metals (main text, lines 55–61), this supports the conclusion that copper and silver inflict distinct antibacterial activity or engage different condition-dependent defense responses.

Given the limited scope of the Observation format and available resources, a detailed mechanistic approach was not pursued and no specific mechanistic hypotheses were formulated nor mechanistic claims stated. Instead, the aim of this study was to report a concise, hypothesis-generating kinetic observation that may motivate future mechanistic investigations in strain- or context-specific settings.

The conclusion that silver-induced lag reflects transient killing followed by regrowth is plausible, but would benefit from complementary assays such as viability staining, single-cell analyses, or persister/tolerant fraction measurements.

We agree that single-cell approaches can in principle provide valuable complementary information. We considered such approaches, especially the aspect of viability staining as it allows simultaneous measurement during growth assays as

well as single cell analysis by flow cytometry (FCM) and is expected to result in less technical variability than cultivation-based viable counts seen on Fig 1C and 1D.

However, validity of fluorescence-based single-cell assays in metal-exposure systems is inherently challenged by metal-induced nucleic acid degradation, fluorescence quenching, and redox interference. Copper exposure is well documented to interact with nucleic acids, cause rapid degradation and to interfere with nucleic-acid–based detection methods (Hong et al., 2012; Kuffel et al., 2021; Manuel et al., 2015; Mathews et al., 2015; Moreno and McCord, 2017; Moshkovich et al., 2021; Sarah L. Warnes et al., 2012; S. L. Warnes et al., 2012; Warnes et al., 2015, 2010; Warnes and Keevil, 2011; Weaver et al., 2010; Yamamoto et al., 2021), while silver can exert similar effects possibly to a lesser extent (Gu et al., 2018; Hossain and Huq, 2002; Hsueh et al., 2015; Mohapatra et al., 2023; Moshkovich et al., 2021). In addition, both copper and silver can distort fluorescence-based readouts through photophysical or chemical interactions with reporters, including FRET that is specifically exploited in metal-sensor development (Bourge et al., 2015; Hötzer et al., 2011; Jensen et al., 2024).

Redox-based vitality indicators face similar limitations, as redox-active metals such as copper can directly affect sensor chemistry or indirectly alter metabolic activity, membrane potential, and electron transport, thereby decoupling fluorescence readouts from survival. For example, redox-based vitality sensors are specifically used to assess silver effects on electron transport chain (Priester et al., 2014). Such limitations are particularly relevant under conditions involving transient or delayed post-exposure killing described by Fanous et al. (Fanous et al., 2025).

With respect to persister or tolerant-fraction estimation during biocidal exposure standard approaches rely on biphasic kill curves and a stable persistence plateau (Balaban et al., 2019; Krewing et al., 2024; Ronneau et al., 2021). In contrast, the silver exposures in Fig 1D produce a U-shaped kill–regrowth dynamic, suggesting potentially overlapping killing and regrowth processes. Under these conditions, quantitative estimation of a persister fraction is not straightforward without resolving single-cell-level temporal dynamics.

Because establishing and validating the necessary metal-specific controls is non-trivial and highly context-dependent, and because comparable controls would be required to enable an unbiased quantitative comparison of copper and silver effects under identical conditions, we did not pursue single-cell fluorescence-based assays in the present study. Addressing these methodological challenges represents an important direction for future work but was beyond the scope of this Observation.

MIC values are central to the framing of "sub-MIC" exposure, yet the manuscript does not clearly detail how MICs were determined under the same experimental conditions or whether MICs remain stable over time. Also, time-kill methodology lacks some critical details

All experiments were conducted as described in Supplementary Note 2. To address the reviewer's comment regarding MIC determination, we added a more detailed description of MIC assessment in the Supplementary Notes and Figures file of the revised manuscript, as indicated below in bold.

Second subculture of E. coli on LB was used to inoculate overnight liquid LB cultures. The stationary culture was 100-fold diluted into fresh LB and grown to exponential phase (OD_{600}=0.5-0.7), then washed twice with cold PBS (4°C, 4400 g, 10 min). Pellets were resuspended in 2× MMM and adjusted to OD_{600} = 0.002 (~10^6 CFU/mL). Inoculum was added to twofold dilution series of $AgNO_3$ or $CuSO_4$ or mixed 1:1 with deionized water and serially diluted in 1× MMM to test inoculum size effects. The final volume of exposures in 96-well suspension culture plates was 150 µl for growth curves and 200 µl for kill curves. Sterile water was pipetted between the wells and plates sealed with parafilm to reduce drying during long incubations.

Growth curves were measured at OD_{600} every 15 min at 37°C with double-orbital mixing (BioTek Synergy H1, Agilent). Blank-corrected data were analyzed with Dashing Growth Curves at https://dashing-growth-curves.ethz.ch/.

Minimal inhibitory concentrations (MIC) of $AgNO_3$ or $CuSO_4$ were determined from growth curves as the lowest concentration at which no time-dependent OD increase was registered during the experiments and verified by no visible growth after 48 h incubation.

For kill curves, exposure plates were incubated at 37°C, 150 rpm in the dark. At each time-point, three concentration series per metal were serially diluted in PBS and drop-plated on LB; 150 µL of undiluted exposures were also spread-plated on 10 cm LB plates for viable counts. Survival was expressed as log_{10}-transformed survivor fraction (post-/pre-exposure viable counts).

With respect to MIC stability, no experiments involving repeated or evolutionary exposures were performed. Under the defined strain, medium, and growth-condition combination used in this study, MIC values were reproducible across independent experiments. If needed, MIC values for shorter time points are directly reflected in the growth and kill kinetics presented in Figure 1.

If additional clarification regarding the time-kill assay is required, we would be happy to provide further detail. Our aim was to conduct the "growth" and "kill" experiments in as similar conditions as possible. Essentially, either OD for growth experiments or CFU/ml for kill experiments was measured from similar exposures. Unfortunately, both cannot be measured simultaneously from the same plate.

Toxicity of Cu and Ag depends strongly on ion speciation and chemical state. Without measurements or controls for free ion activity or complexation, reproducibility and cross-study comparability are uncertain.

> We agree that the toxicity of metal-based antimicrobials is strongly influenced by ion speciation and the chemical environment. As discussed in Supplementary Note 1 (section "*Exposure medium selection")*, metal speciation in biological systems is highly dynamic and depends on both abiotic factors (*e.g.,* medium composition, pH, chloride content) and biotic components (*e.g.,* biomolecules and dead cell matter), which continuously modulate metal bioavailability during exposure.
>
> In this study, a minimally complexing, MOPS-buffered defined medium was selected to reduce pH-driven and medium-dependent biases while allowing direct comparison of copper and silver effects under similar conditions. While direct measurement of free ion activity can be informative in simplified chemical systems, reliance on free ion concentration alone represents an oversimplification in dynamic biological exposures, where metal complexes may remain bioavailable and contribute to toxicity via alternative uptake routes. *E.g* metal-amino-acid complexes can hitchhike amino acid transporters and gain an uptake route due to which disruption of amino acid transporters can decrease toxic metal uptake as in the case of copper and silver (Rosenberg et al., 2025) or selenium (Deutch et al., 2014) and cystine transport via YdjN.
>
> Our experimental design therefore prioritized internal consistency and comparability of growth and kill kinetics between copper and silver rather than absolute quantification of free ion activity. The limitations associated with speciation and medium dependence are explicitly acknowledged, and the chosen conditions reflect a compromise that is both experimentally tractable and biologically relevant within the scope of this Observation.

Growth and killing trends are primarily interpreted visually. Explicit statistical modeling of growth rates, lag times, killing rates, and effect sizes would strengthen the conclusions.

> Growth and killing dynamics were not interpreted solely based on visual inspection. Growth parameters, including lag phase duration, doubling time, and biomass yield, were quantitatively extracted from growth curves and statistically analyzed as described in Supplementary Note 2. These analyses are summarized in Supplementary Figures S1 and S2 and support the reported dose-dependent effects of copper and silver on growth kinetics.
>
> Visual presentation of representative growth and kill curves in Figure 1 was chosen to convey the overall kinetic behavior underlying the observation, while detailed parameterization and statistical testing were provided as supplementary information.

With respect to kill kinetics, we agree that rate-based modeling can be informative. However, the rapid initial killing followed by regrowth observed under silver exposure results in U-shaped kill–growth dynamics, complicating rate estimation without resolving whether killing and regrowth occur sequentially or overlap temporally. Addressing this would require experimental designs that differ from those used to maintain comparability between growth and kill assays and was therefore beyond the scope of the present study.

Claims regarding antimicrobial testing practices and risk assessment would be stronger if supported by at least one additional strain or species.

We agree that extending the analysis to additional strains or species would strengthen generalizability. However, the present study was intentionally designed and submitted as an *Observation* to report a focused kinetic phenomenon rather than to provide broad strain- or species-level validation.

As discussed in Supplementary Note 1 (sections *"Strain and inoculum selection"* and *"Re-growth conditions")*, strain selection and growth conditions represent acknowledged limitations of the study. The objective here was to demonstrate that sub-MIC copper and silver exposures can produce fundamentally different growth and kill kinetics that are not captured by standard endpoint assays, even within a single, well-defined experimental system.

We view this observation as hypothesis-generating and as motivation for future strain- and context-specific investigations, rather than as a comprehensive assessment of antimicrobial risk across taxa.

The manuscript draws broad implications for antimicrobial product evaluation and resistance development that may not be fully supported by the relatively compact dataset presented. While the manuscript raises an important conceptual point and presents solid preliminary data, its current form may not meet the evidentiary and scope expectations for a full research article. Substantial revision-particularly expanding experimental breadth, strengthening mechanistic or evolutionary support, and tightening the discussion-would be required.

We agree that the dataset presented is compact and that the manuscript is not intended to meet the evidentiary breadth, mechanistic depth, or experimental scope expected of a full Research Article. The study was therefore intentionally submitted as an *Observation* to report a focused kinetic finding supported by a limited but well-defined dataset, rather than to provide comprehensive mechanistic, evolutionary, or product-level validation.

Accordingly, the discussion of antimicrobial testing practices, tolerance, resistance, and risk assessment is framed to highlight plausible implications of the observed

kinetic differences between copper and silver, rather than to draw definitive or generalizable conclusions. We have aimed to clearly delimit the scope of the study, acknowledge its limitations, and present the findings as hypothesis-generating. Within the expectations of the Observation format, we believe that the current data sufficiently supports the central conceptual point that reliance on static endpoint assays such as MIC can obscure biologically relevant sub-MIC toxicity dynamics.

Reviewer #2 (Comments for the Author):

In this manuscript Rosenberg, et al examine growth behavior of E. coli in the presence of sub inhibitory concentrations of silver and copper, two well-known metals with antimicrobial properties. Overall the manuscript was well written and provided an interesting data set, described and discussed well by the authors. Included below are a few comments that the authors may wish to address to further strengthen the manuscript and add clarity for readers.
1. Figure one C/D was more difficult to interpret the significant of the range "whiskers". Not sure if some spots they were overlapping, but I struggled with them particularly at the right side of the two plots.

We thank the reviewer for this comment. In Figure 1C and 1D, viable counts are presented as mean ± standard deviation from three biological replicates, with the primary aim of illustrating temporal changes in survival rather than statistically comparing dose-dependent effects at individual time points.

To improve clarity, we added a bar-graph representation of the same survival data as Supplementary Figure S5, which facilitates interpretation of variability across conditions. The relatively large variability observed for longer exposures or higher silver concentrations reflects inherent biological and technical variability associated with cultivation-based viable count measurements, particularly under conditions of substantial killing. The raw data underlying these figures are provided in the Supplementary Data file.

2. Remove e.g. in line 110 or restructure the sentence.

We have rephrased the indicated sentence to improve readability, as suggested.

On the other hand, silver resistance is a well-known challenge necessitating combined treatment strategies, e.g. in burn wound care (17, 18).

3. I think the conclusion line could use further argument/discussion for justification or should be more directly correlated with the data in this observation manuscript.

We have revised the concluding sentence to more explicitly link the broader implications to the kinetic observations presented in this study. The revised wording emphasizes that the conclusions are based on the demonstrated limitations of endpoint MIC assays in capturing transient killing and regrowth dynamics, rather than on generalized claims about resistance development (addition in bold):

> *In conclusion, our observations highlight new and known challenges in assessing antimicrobial characteristics by using wide-spread endpoint assays such as MIC* ***that mask toxicity kinetics*** *to make decisions guiding the design and risk assessment of antimicrobial formulations in different fields of application.*

Aboubakr, H.A., Sharafeldin, T.A., Goyal, S.M., 2021. Stability of SARS-CoV-2 and other coronaviruses in the environment and on common touch surfaces and the influence of climatic conditions: A review. Transbound. Emerg. Dis. 68, 296–312. https://doi.org/10.1111/tbed.13707

Balaban, N.Q., Helaine, S., Lewis, K., Ackermann, M., Aldridge, B., Andersson, D.I., Brynildsen, M.P., Bumann, D., Camilli, A., Collins, J.J., Dehio, C., Fortune, S., Ghigo, J.-M., Hardt, W.-D., Harms, A., Heinemann, M., Hung, D.T., Jenal, U., Levin, B.R., Michiels, J., Storz, G., Tan, M.-W., Tenson, T., Van Melderen, L., Zinkernagel, A., 2019. Definitions and guidelines for research on antibiotic persistence. Nat. Rev. Microbiol. 17, 441–448. https://doi.org/10.1038/s41579-019-0196-3

Blanco Massani, M., Klumpp, J., Widmer, M., Speck, C., Nisple, M., Lehmann, R., Schuppler, M., 2018. Chromosomal Sil system contributes to silver resistance in E. coli ATCC 8739. BioMetals 31, 1101–1114. https://doi.org/10.1007/s10534-018-0143-1

Bourge, M., Fort, C., Soler, M., Satiat-Jeunemaître, B., Brown, S.C., 2015. A pulse-chase strategy combining click-EdU and photoconvertible fluorescent reporter: tracking Golgi protein dynamics during the cell cycle. New Phytol. 205, 938–950. https://doi.org/10.1111/nph.13069

Deutch, C.E., Spahija, I., Wagner, C.E., 2014. Susceptibility of *Escherichia coli* to the toxic L-proline analogue L-selenaproline is dependent on two L-cystine transport systems. J. Appl. Microbiol. 117, 1487–1499. https://doi.org/10.1111/jam.12623

Fanous, J., Claudi, B., Tripathi, V., Li, J., Goormaghtigh, F., Bumann, D., 2025. Limited impact of Salmonella stress and persisters on antibiotic clearance. Nature 639, 181–189. https://doi.org/10.1038/s41586-024-08506-6

Gu, L., Saran, R., Yan, W., Huang, P.-J.J., Wang, S., Lyu, M., Liu, J., 2018. Reselection Yielding a Smaller and More Active Silver-Specific DNAzyme. ACS Omega 3, 15174–15181. https://doi.org/10.1021/acsomega.8b02039

Hong, R., Kang, T.Y., Michels, C.A., Gadura, N., 2012. Membrane Lipid Peroxidation in Copper Alloy-Mediated Contact Killing of Escherichia coli. Appl. Environ. Microbiol. 78, 1776–1784. https://doi.org/10.1128/AEM.07068-11

Hossain, Z., Huq, F., 2002. Studies on the interaction between Ag+ and DNA. J. Inorg. Biochem. 91, 398–404. https://doi.org/10.1016/S0162-0134(02)00454-3

Hosseini, M., Chin, A.W.H., Behzadinasab, S., Poon, L.L.M., Ducker, W.A., 2021. Cupric Oxide Coating That Rapidly Reduces Infection by SARS-CoV-2 via Solids. ACS Appl. Mater. Interfaces 13, 5919–5928. https://doi.org/10.1021/acsami.0c19465
Hötzer, B., Ivanov, R., Altmeier, S., Kappl, R., Jung, G., 2011. Determination of Copper(II) Ion Concentration by Lifetime Measurements of Green Fluorescent Protein. J. Fluoresc. 21, 2143–2153. https://doi.org/10.1007/s10895-011-0916-1
Hsueh, Y.-H., Lin, K.-S., Ke, W.-J., Hsieh, C.-T., Chiang, C.-L., Tzou, D.-Y., Liu, S.-T., 2015. The Antimicrobial Properties of Silver Nanoparticles in Bacillus subtilis Are Mediated by Released Ag+ Ions. PLOS ONE 10, e0144306. https://doi.org/10.1371/journal.pone.0144306
Jensen, G.C., Janis, M.K., Nguyen, H.N., David, O.W., Zastrow, M.L., 2024. Fluorescent Protein-Based Sensors for Detecting Essential Metal Ions across the Tree of Life. ACS Sens. 9, 1622–1643. https://doi.org/10.1021/acssensors.3c02695
Krewing, M., Mönch, E., Bolten, A., Niesalla, H., 2024. Resistance or tolerance? Highlighting the need for precise terminology in the field of disinfection. J. Hosp. Infect. 150, 51–60. https://doi.org/10.1016/j.jhin.2024.05.006
Kuffel, A., Gray, A., Daeid, N.N., 2021. Impact of metal ions on PCR inhibition and RT-PCR efficiency. Int. J. Legal Med. 135, 63–72. https://doi.org/10.1007/s00414-020-02363-4
Manuel, C.S., Moore, M.D., Jaykus, L.A., 2015. Destruction of the Capsid and Genome of GII.4 Human Norovirus Occurs during Exposure to Metal Alloys Containing Copper. Appl. Environ. Microbiol. 81, 4940–4946. https://doi.org/10.1128/AEM.00388-15
Mathews, S., Kumar, R., Solioz, M., 2015. Copper Reduction and Contact Killing of Bacteria by Iron Surfaces. Appl. Environ. Microbiol. 81, 6399–6403. https://doi.org/10.1128/AEM.01725-15
Mohapatra, B., Mohapatra, S., Sharma, N., 2023. Biosynthesized Ag–ZnO nanohybrids exhibit strong antibacterial activity by inducing oxidative stress. Ceram. Int. 49, 20218–20233. https://doi.org/10.1016/j.ceramint.2023.03.146
Moreno, L.I., McCord, B.R., 2017. Understanding metal inhibition: The effect of copper (Cu 2+) on DNA containing samples. Forensic Chem. 4, 89–95. https://doi.org/10.1016/j.forc.2017.03.005
Moshkovich, L., Saphier, O., Popov, S., Shotland, Y., Silberstein, E., Saphier, M., 2021. Monovalent Copper and Silver Ions Block DNA Polymerase Chain Reaction. https://doi.org/10.21203/rs.3.rs-543661/v1
Priester, J.H., Singhal, A., Wu, B., Stucky, G.D., Holden, P.A., 2014. Integrated approach to evaluating the toxicity of novel cysteine-capped silver nanoparticles to Escherichia coli and Pseudomonas aeruginosa. The Analyst 139, 954–963. https://doi.org/10.1039/C3AN01648J
Ronneau, S., Hill, P.W., Helaine, S., 2021. Antibiotic persistence and tolerance: not just one and the same. Curr. Opin. Microbiol. 64, 76–81. https://doi.org/10.1016/j.mib.2021.09.017
Rosenberg, M., Park, S., Umerov, S., Ivask, A., 2025. Experimental evolution of *Escherichia coli* on semi-dry silver, copper, stainless steel, and glass surfaces. Microbiol. Spectr. 13, e02173-24. https://doi.org/10.1128/spectrum.02173-24
Warnes, S. L., Caves, V., Keevil, C.W., 2012. Mechanism of copper surface toxicity in *Escherichia coli* O157:H7 and *Salmonella* involves immediate membrane depolarization followed by slower rate of DNA destruction which differs from that

observed for Gram-positive bacteria. Environ. Microbiol. 14, 1730–1743. https://doi.org/10.1111/j.1462-2920.2011.02677.x

Warnes, S.L., Green, S.M., Michels, H.T., Keevil, C.W., 2010. Biocidal Efficacy of Copper Alloys against Pathogenic Enterococci Involves Degradation of Genomic and Plasmid DNAs. Appl. Environ. Microbiol. 76, 5390–5401. https://doi.org/10.1128/AEM.03050-09

Warnes, Sarah L., Highmore, C.J., Keevil, C.W., 2012. Horizontal Transfer of Antibiotic Resistance Genes on Abiotic Touch Surfaces: Implications for Public Health. mBio 3, e00489-12. https://doi.org/10.1128/mBio.00489-12

Warnes, S.L., Keevil, C.W., 2011. Mechanism of Copper Surface Toxicity in Vancomycin-Resistant Enterococci following Wet or Dry Surface Contact. Appl. Environ. Microbiol. 77, 6049–6059. https://doi.org/10.1128/AEM.00597-11

Warnes, S.L., Summersgill, E.N., Keevil, C.W., 2015. Inactivation of Murine Norovirus on a Range of Copper Alloy Surfaces Is Accompanied by Loss of Capsid Integrity. Appl. Environ. Microbiol. 81, 1085–1091. https://doi.org/10.1128/AEM.03280-14

Weaver, L., Noyce, J.O., Michels, H.T., Keevil, C.W., 2010. Potential action of copper surfaces on meticillin-resistant Staphylococcus aureus: Action of copper surfaces on MRSA. J. Appl. Microbiol. 109, 2200–2205. https://doi.org/10.1111/j.1365-2672.2010.04852.x

Yamamoto, A., Tanaka, S., Ohishi, K., 2021. Quantitative Evaluation of Nucleic Acid Degradability of Copper Alloy Surfaces and Its Correlation to Antibacterial Activity. Antibiot. Basel Switz. 10, 1439. https://doi.org/10.3390/antibiotics10121439

Re: Spectrum03512-25R1 (Distinct sub-MIC kill kinetics of Cu and Ag in *Escherichia coli*)

Dear Dr. Merilin Rosenberg:

Thank you for the privilege of reviewing your work. Below you will find my comments, instructions from the Spectrum editorial office, and the reviewer comments.

Please return the manuscript within 60 days; if you cannot complete the modification within this time period, please contact me. If you do not wish to modify the manuscript and prefer to submit it to another journal, notify me immediately so that the manuscript may be formally withdrawn from consideration by Spectrum.

Revision Guidelines
To submit your modified manuscript, log into the submission site at https://spectrum.msubmit.net/cgi-bin/main.plex. Go to Author Tasks and click the appropriate manuscript title to begin. The information you entered when you first submitted the paper will be displayed; update this as necessary. Note the following requirements:

• Upload point-by-point responses to the issues raised by the reviewers in a file named "Response to Reviewers," NOT in your cover letter.
• Upload a compare copy of the manuscript (without figures) as a "Marked-Up Manuscript" file.
• Upload a clean .DOC/.DOCX version of the revised manuscript and remove the previous version.
• Each figure must be uploaded as a separate, editable, high-resolution file (TIFF or EPS preferred), and any multipanel figures must be assembled into one file.
• Any supplemental material intended for posting by ASM should be uploaded with their legends separate from the main manuscript. You can combine all supplemental material into one file (preferred) or split it into a maximum of 10 files with all associated legends included.

For complete guidelines on revision requirements, see our Submission and Review Process webpage. Submission of a paper that does not conform to guidelines may delay acceptance of your manuscript.

Data availability: ASM policy requires that data be available to the public upon online posting of the article, so please verify all links to sequence records, if present, and make sure that each number retrieves the full record of the data. If a new accession number is not linked or a link is broken, provide Spectrum production staff with the correct URL for the record. If the accession numbers for new data are not publicly accessible before the expected online posting of the article, publication may be delayed; please contact production staff (Spectrum@asmusa.org) immediately with the expected release date.

Publication Fees: For information on publication fees and which article types are subject to charges, visit our website. If your manuscript is accepted for publication and any fees apply, you will be contacted separately about payment during the production process; please follow the instructions in that e-mail. Arrangements for payment must be made before your article is published.

ASM Membership: Corresponding authors may join or renew ASM membership to obtain discounts on publication fees. Need to upgrade your membership level? Please contact Customer Service at Service@asmusa.org.

The ASM Journals program strives for constant improvement in our submission and publication process. Please tell us how we can improve your experience by taking this quick Author Survey.

Thank you for submitting your paper to Spectrum.

Sincerely,
Mor Lurie-Weinberger
Editor
Microbiology Spectrum

Reviewer #3 (Comments for the Author):

The manuscript presents interesting and valuable data on the distinct sub-MIC kill kinetics of copper and silver in E. coli. The observation that copper primarily inhibits growth while silver causes transient killing followed by regrowth is a significant contribution. However, a major conceptual leap is made from these short-term kinetic observations to conclusions about the differential selection for antimicrobial tolerance and resistance.

While the authors suggest that these kinetic differences could select for tolerance (in the case of silver) or resistance (in the case of copper), the current dataset does not provide any direct evidence for this evolutionary outcome. The experiments are short-term (48h) and do not demonstrate that the observed phenotypes (e.g., survival without growth for silver, sustained slow growth for copper) lead to heritable changes in the bacterial population. The terms "select for" imply an evolutionary process that has not been experimentally validated here. Statements in the Abstract and main text (e.g., "Our results suggest that short-term survival... is sufficient for selective advantage") overreach the data, as they do not demonstrate that this survival actually translates into a long-term competitive advantage that shapes the population's evolutionary trajectory under repeated or prolonged exposure.

To strengthen the manuscript, the authors should revise the language throughout, especially in the Abstract and Discussion, to frame the potential for differential selection as a hypothesis or an implication stemming from their kinetic data, rather than a demonstrated finding. Use more cautious phrasing such as "may have implications for the development of tolerance" or "suggests a potential for differential selective pressures."

Reviewer #4 (Comments for the Author):

The present study "Distinct sub-MIC kill kinetics of Cu and Ag in Escherichia coli" is a basic observational study with several shortcomings:

1. The study relied exclusively on a single E. coli strain (BW25113), limiting generalizability. Antimicrobial responses vary between strains, and without testing multiple isolates, the observed differential kinetics cannot be confidently attributed to copper versus silver. Including a gram-positive bacterium would have provided useful comparative information.

2. The time-kill data sampling frequency appears insufficient to characterise the transient killing claimed for silver. The critical period immediately after exposure (first 30-60 min) lacks clarity, making it difficult to distinguish true killing from growth rate effects or sampling artifacts.

3. Using 48-hour MIC values as reference points for sub-MIC concentrations is unconventional. Standard MIC determinations are read at 16-20 h, and extending to 48 h may introduce confounding factors like nutrient depletion or pH changes that could alter the apparent MIC.

4. The study identifies phenotypic differences but provides no mechanistic data explaining why copper and silver produce different killing kinetics. Without examining uptake, efflux, or molecular targets, the observations remain descriptive rather than explanatory.

5. Without experimental evolution or long-term exposure experiments, extrapolating from short-term killing to long-term resistance outcomes is a logical leap. The claim that silver poses "higher risk for tolerance and resistance" is speculative without direct comparative evolution data.

6. The defined laboratory conditions bear little resemblance to real-world scenarios where these metals are used. Factors like organic matter, biofilms, and microbial communities would likely alter the observed kinetics and should be considered.

7. The study would be strengthened by complementary techniques such as flow cytometry with live/dead staining or microscopy to observe single-cell behaviour rather than relying solely on population-level plating.

These limitations suggest that while the study presents an interesting observation, the methodological approach is limited and the conclusions exceed what the data can support.

Response to the reviewers

We thank the reviewers for their careful evaluation of our manuscript and for the constructive comments provided. We would like to emphasize that this study was intentionally submitted as an *Observation* (short description of research results of interest to the broad microbiology community: maximum 1,200 words with a maximum of 2 figures and 25 references) and not a full Research Article, to report a focused and potentially impactful kinetic observation rather than to provide a comprehensive mechanistic analysis. Accordingly, the scope of the work is limited to a single well-defined experimental system and a compact dataset designed to highlight distinct sub-MIC growth and kill kinetics of copper and silver that are not captured by widely used endpoint assays.

Below, reviewer comments (in **black**) from both first revision (Reviewers 1 and 2) and second revision (Reviewers 3 and 4) are addressed one-by-one by the authors (in **blue**) with cited text indented and in *Italic*. All cited references are listed at the end of the document.

Reviewer #3 (Comments for the Author):

The manuscript presents interesting and valuable data on the distinct sub-MIC kill kinetics of copper and silver in E. coli. The observation that copper primarily inhibits growth while silver causes transient killing followed by regrowth is a significant contribution. However, a major conceptual leap is made from these short-term kinetic observations to conclusions about the differential selection for antimicrobial tolerance and resistance.

While the authors suggest that these kinetic differences could select for tolerance (in the case of silver) or resistance (in the case of copper), the current dataset does not provide any direct evidence for this evolutionary outcome. The experiments are short-term (48h) and do not demonstrate that the observed phenotypes (e.g., survival without growth for silver, sustained slow growth for copper) lead to heritable changes in the bacterial population. The terms "select for" imply an evolutionary process that has not been experimentally validated here. Statements in the Abstract and main text (e.g., "Our results suggest that short-term survival... is sufficient for selective advantage") overreach the data, as they do not demonstrate that this survival actually translates into a long-term competitive advantage that shapes the population's evolutionary trajectory under repeated or prolonged exposure.

To strengthen the manuscript, the authors should revise the language throughout, especially in the Abstract and Discussion, to frame the potential for differential selection as a hypothesis or an implication stemming from their kinetic data, rather than a demonstrated finding. Use more cautious phrasing such as "may have implications for the development of tolerance" or "suggests a potential for differential selective pressures."

> We thank the reviewer for highlighting this important conceptual point. We agree that the current dataset does not directly demonstrate evolutionary outcomes such as heritable tolerance or resistance. The intent of our statements was to interpret the

observed kinetic differences in the context of established frameworks of tolerance and resistance development, rather than to claim experimentally validated selection.

To address this, we have revised and rephrased the whole manuscript text to avoid deterministic language such as “select for” and instead frame these interpretations as hypotheses or potential implications of the observed kinetic behaviors. Specifically, we now emphasize that the transient survival observed under silver exposure and sustained growth under copper exposure *may represent conditions that could favor different adaptive trajectories*, without asserting that such outcomes were demonstrated in this study.

Reviewer #4 (Comments for the Author):
The present study "Distinct sub-MIC kill kinetics of Cu and Ag in Escherichia coli" is a basic observational study with several shortcomings:

1. The study relied exclusively on a single E. coli strain (BW25113), limiting generalizability. Antimicrobial responses vary between strains, and without testing multiple isolates, the observed differential kinetics cannot be confidently attributed to copper versus silver. Including a gram-positive bacterium would have provided useful comparative information.

We agree that the use of a single strain limits generalizability. As noted in Supplementary Note 1, strain- and species-specific variability in metal susceptibility is an important consideration. The present study was intentionally designed as a controlled single-system observation to isolate and demonstrate kinetic differences between copper and silver under defined conditions. We have further clarified this limitation in the main text and now explicitly state that the findings should be interpreted as system-specific and hypothesis-generating, warranting validation across additional strains, species and conditions in future work.

2. The time-kill data sampling frequency appears insufficient to characterise the transient killing claimed for silver. The critical period immediately after exposure (first 30-60 min) lacks clarity, making it difficult to distinguish true killing from growth rate effects or sampling artifacts.

We agree that higher temporal resolution during the initial exposure period could further refine characterization of early killing dynamics. In the current design, sampling intervals were selected to balance temporal resolution with technical reproducibility across multiple conditions and replicates. Additionally, the rapid initial killing followed by regrowth observed under silver exposure results in U-shaped kill–growth dynamics, complicating rate estimation without resolving whether killing and regrowth occur sequentially or overlap temporally. Addressing this would require experimental designs that differ from those used here to maintain comparability

between growth and kill assays and was therefore beyond the scope of the present study. Importantly, the observed >4-log reduction in viable counts within the early time points (Fig. 1D) robustly supports the occurrence of rapid killing followed by regrowth, even if finer temporal structure in early exposures is not fully resolved. We have added a statement acknowledging this limitation.

> *"Although much higher temporal resolution in early exposure would be needed to calculate and compare kill rates across concentrations, rapid up to >4-log killing followed by regrowth displaying dose-dependently increased lag phase duration under antimicrobial exposures as in case of silver in Fig 1D and 1B, could be primarily attributable to sub-MIC killing and reduced viable cell count at resumption of growth. "*

3. Using 48-hour MIC values as reference points for sub-MIC concentrations is unconventional. Standard MIC determinations are read at 16-20 h, and extending to 48 h may introduce confounding factors like nutrient depletion or pH changes that could alter the apparent MIC.

We acknowledge that standardized antibiotic MIC determinations according to CLSI and EUCAST guidelines are read after 16–20 h of incubation for non-fastidious bacteria such as *E. coli*. In the present study, MIC values were not used for clinical breakpoints but as operational reference points within a kinetic experimental framework. Specifically, MIC was defined based on the absence of detectable growth over 48 h to ensure consistency with the extended growth and kill kinetics measurements and to capture delayed growth recovery effects observed under metal exposure conditions. Importantly, MIC values corresponding to both standard (16-20 h) and extended (48 h) timepoints are now explicitly reported (Fig. 1), allowing direct comparison to conventional definitions. We have clarified this distinction in the manuscript to avoid confusion between standardized clinical antibiotic MIC determinations and experimentally defined MIC values used for kinetic analyses.

We have clarified this rationale and now explicitly state MIC in both time points of interest (18 h and 48 h) in the legend of Fig 1 and added the following explanation to Supplementary Note 2:

> *"MIC values of 4 mM and 8 mM Cu after 18 and 48 h, respectively, or 1.95 µM Ag after both 18 h and 48 h are stated in the results to represent 16-20 h standard condition for MIC determination as well as operational definitions aligned with the experimental design of the study."*

4. The study identifies phenotypic differences but provides no mechanistic data explaining why copper and silver produce different killing kinetics. Without examining uptake, efflux, or molecular targets, the observations remain descriptive rather than explanatory.

We agree that the study does not provide mechanistic resolution. As outlined in the manuscript, this was a deliberate scope decision aligned with the short Observation format of the publication type. The aim was to report a reproducible kinetic phenomenon rather than to resolve underlying molecular mechanisms. We have clarified in the revised manuscript that the observed differences are interpreted at the level of phenotypic outcomes (growth inhibition vs. transient killing), and that mechanistic dissection—such as metal uptake, intracellular speciation, or target damage—remains an important direction for future studies.

> *"Under the conditions tested, silver caused transient killing followed by regrowth of survivors, whereas copper caused sustained growth inhibition with minimal early killing. These phenotypic differences now warrant future mechanistic studies of metal uptake, intracellular speciation, damage, and defense responses."*

5. Without experimental evolution or long-term exposure experiments, extrapolating from short-term killing to long-term resistance outcomes is a logical leap. The claim that silver poses "higher risk for tolerance and resistance" is speculative without direct comparative evolution data.

We agree that extrapolation from short-term kinetics to long-term evolutionary outcomes requires caution. As also noted by Reviewer #3, we have systematically revised all relevant statements to remove what can be read as claims regarding selection for tolerance or resistance. The text now frames these connections as hypotheses grounded in the observed phenotypic dynamics and existing literature, rather than implying conclusions supported by experimental evolution data.

6. The defined laboratory conditions bear little resemblance to real-world scenarios where these metals are used. Factors like organic matter, biofilms, and microbial communities would likely alter the observed kinetics and should be considered.

We agree that real-world environments involve additional complexity, including ambient conditions, organic and inorganic soiling, biofilms, and microbial communities, which can substantially alter metal bioavailability and antimicrobial effects. The present study was designed to isolate kinetic differences under controlled conditions using a defined medium to minimize confounding variables. We have now explicitly acknowledged that the observed kinetics may differ in more complex environments and that extending these findings to applied settings requires further investigation under environmentally relevant conditions. Method description in Supplementary Note 1 was extended with the following:

> *"In complex environments such as biofilms or organic-rich matrices, metal speciation and microbial interactions may substantially alter the observed kinetics. However, proof-of-principle research must first study biological effects*

in controlled conditions with minimal confounding factors. In addition to strain and growth phase selection, the growth medium to our best knowledge least interfering the metal-microbe interactions was selected as described below."

7. The study would be strengthened by complementary techniques such as flow cytometry with live/dead staining or microscopy to observe single-cell behaviour rather than relying solely on population-level plating.

We agree that single-cell approaches could provide valuable complementary insights. However, fluorescence- and redox-based single-cell assays in copper and silver exposure systems are methodologically difficult to validate and compare quantitatively because of metal-dependent interference with nucleic acids, fluorophores, and reporter chemistry. For this reason, such approaches were not pursued within the scope of the present Observation. A more detailed discussion on the same subject is provided in our response to Reviewer #1 below.

These limitations suggest that while the study presents an interesting observation, the methodological approach is limited and the conclusions exceed what the data can support.

Reviewer #1 (Comments for the Author):

The manuscript addresses an important and timely issue: the limitations of endpoint MIC-based assays for evaluating antimicrobial effects of metals such as copper and silver. The study highlights how sub-MIC exposures can produce complex growth and killing dynamics that may be obscured by standard assays. The work is clearly written, the experimental logic is sound, and the question is relevant to antimicrobial stewardship and product evaluation. However, while the findings are interesting, the current version of the manuscript has several limitations in scope, experimental depth, and evidentiary support that, taken together, may prevent acceptance as a full research article in its present form.

Below, I outline specific concerns that would need to be addressed.
The study is based on a single laboratory strain (Escherichia coli BW25113). Given the known strain-specific variability in metal tolerance and resistance mechanisms, the generality of the conclusions is uncertain.

We agree that metal tolerance and resistance can vary across *E. coli* strains, growth phases, physiological states, and between species. These limitations, including the choice of strain and growth phase, are explicitly acknowledged and discussed in Supplementary Note 1 (section *"Strain and inoculum selection")*.

The kinetic effects reported here were initially observed in *Escherichia coli* ATCC 8739 strain. However, this strain rapidly acquires reduced silver and copper susceptibility due to stochastic mutations in the chromosomal *sil* locus, as demonstrated previously by us and others (Blanco Massani et al., 2018; Rosenberg et al., 2025). This instability

precludes its use for robust quantitative analyses. We therefore selected the stable K-12 derivative BW25113 as a model strain, which also enables potential future functional follow-up using established genetic resources such as the KEIO mutant collection.

While the discussion acknowledges uncertainty in copper and silver toxicity mechanisms, no experiments are included to test specific hypotheses (e.g., oxidative stress, membrane damage, metal uptake or efflux).

We acknowledge that a broad and partially overlapping arsenal of toxicity and defense mechanisms has been described for both copper and silver (main text, lines 46–55). Unlike most antibiotics, metals exert multimechanistic effects, and more often than not the question is not what happened as a population-level sum of events during the exposure but rather what ultimately caused the irreversible changes or overwhelmed the bacterial defenses that eventually lead to cell death and what is collateral or even post-mortem damage. Consequently, detection of *e.g.* membrane damage, envelope stress, loss of proton motive force, DNA damage, or oxidative stress responses does not establish any single mechanism as the primary cause of cell death.

In the context of the present study, population-level mechanistic assays would also be poorly suited to address the key observation in Fig. 1D, where survival of a minute subpopulation following transient silver-induced killing determines the apparent growth delay. Such assays would primarily describe the majority of cells that did not survive the exposure rather than the properties enabling regrowth. Nevertheless, the kinetic data allow one mechanistic inference: copper causes sustained growth inhibition (Fig 1A), whereas the measurable antibacterial effect of silver is transient (Fig 1B). Together with prior evidence for exposure-condition–dependent toxicity of these metals (main text, lines 55–61), this supports the conclusion that copper and silver inflict distinct antibacterial activity or engage different condition-dependent defense responses.

Given the limited scope of the Observation format and available resources, a detailed mechanistic approach was not pursued and no specific mechanistic hypotheses were formulated nor mechanistic claims stated. Instead, the aim of this study was to report a concise, hypothesis-generating kinetic observation that may motivate future mechanistic investigations in strain- or context-specific settings.

The conclusion that silver-induced lag reflects transient killing followed by regrowth is plausible, but would benefit from complementary assays such as viability staining, single-cell analyses, or persister/tolerant fraction measurements.

We agree that single-cell approaches can in principle provide valuable complementary information. We considered such approaches, especially the aspect of viability staining as it allows simultaneous measurement during growth assays as well as single cell analysis by flow cytometry (FCM) and is expected to result in less technical variability than cultivation-based viable counts seen on Fig 1C and 1D.

However, validity of fluorescence-based single-cell assays in metal-exposure systems is inherently challenged by metal-induced nucleic acid degradation, fluorescence quenching, and redox interference. Copper exposure is well documented to interact with nucleic acids, cause rapid degradation and to interfere with nucleic-acid–based detection methods (Hong et al., 2012; Kuffel et al., 2021; Manuel et al., 2015; Mathews et al., 2015; Moreno and McCord, 2017; Moshkovich et al., 2021; Sarah L. Warnes et al., 2012; S. L. Warnes et al., 2012; Warnes et al., 2015, 2010; Warnes and Keevil, 2011; Weaver et al., 2010; Yamamoto et al., 2021), while silver can exert similar effects possibly to a lesser extent (Gu et al., 2018; Hossain and Huq, 2002; Hsueh et al., 2015; Mohapatra et al., 2023; Moshkovich et al., 2021). In addition, both copper and silver can distort fluorescence-based readouts through photophysical or chemical interactions with reporters, including FRET that is specifically exploited in metal-sensor development (Bourge et al., 2015; Hötzer et al., 2011; Jensen et al., 2024).

Redox-based vitality indicators face similar limitations, as redox-active metals such as copper can directly affect sensor chemistry or indirectly alter metabolic activity, membrane potential, and electron transport, thereby decoupling fluorescence readouts from survival. For example, redox-based vitality sensors are specifically used to assess silver effects on electron transport chain (Priester et al., 2014). Such limitations are particularly relevant under conditions involving transient or delayed post-exposure killing described by Fanous et al. (Fanous et al., 2025).

With respect to persister or tolerant-fraction estimation during biocidal exposure standard approaches rely on biphasic kill curves and a stable persistence plateau (Balaban et al., 2019; Krewing et al., 2024; Ronneau et al., 2021). In contrast, the silver exposures in Fig 1D produce a U-shaped kill–regrowth dynamic, suggesting potentially overlapping killing and regrowth processes. Under these conditions, quantitative estimation of a persister fraction is not straightforward without resolving single-cell-level temporal dynamics.

Because establishing and validating the necessary metal-specific controls is non-trivial and highly context-dependent, and because comparable controls would be required to enable an unbiased quantitative comparison of copper and silver effects under identical conditions, we did not pursue single-cell fluorescence-based assays in the present study. Addressing these methodological challenges represents an important direction for future work but was beyond the scope of this Observation.

MIC values are central to the framing of "sub-MIC" exposure, yet the manuscript does not clearly detail how MICs were determined under the same experimental conditions or whether MICs remain stable over time. Also, time-kill methodology lacks some critical details

All experiments were conducted as described in Supplementary Note 2. To address the reviewer's comment regarding MIC determination, we added a more detailed description of MIC assessment in the Supplementary Notes and Figures file of the revised manuscript, as indicated below in bold.

Second subculture of E. coli on LB was used to inoculate overnight liquid LB cultures. The stationary culture was 100-fold diluted into fresh LB and grown to exponential phase (OD_{600}=0.5-0.7), then washed twice with cold PBS (4°C, 4400 g, 10 min). Pellets were resuspended in 2× MMM and adjusted to OD_{600} = 0.002 (~10^6 CFU/mL). Inoculum was added to twofold dilution series of $AgNO_3$ or $CuSO_4$ or mixed 1:1 with deionized water and serially diluted in 1× MMM to test inoculum size effects. The final volume of exposures in 96-well suspension culture plates was 150 µl for growth curves and 200 µl for kill curves. Sterile water was pipetted between the wells and plates sealed with parafilm to reduce drying during long incubations.

Growth curves were measured at OD_{600} every 15 min at 37°C with double-orbital mixing (BioTek Synergy H1, Agilent). Blank-corrected data were analyzed with Dashing Growth Curves at https://dashing-growth-curves.ethz.ch/.

Minimal inhibitory concentrations (MIC) of $AgNO_3$ or $CuSO_4$ were determined from growth curves as the lowest concentration at which no time-dependent OD increase was registered during the experiments and verified by no visible growth after 48 h incubation.

For kill curves, exposure plates were incubated at 37°C, 150 rpm in the dark. At each time-point, three concentration series per metal were serially diluted in PBS and drop-plated on LB; 150 µL of undiluted exposures were also spread-plated on 10 cm LB plates for viable counts. Survival was expressed as log_{10}-transformed survivor fraction (post-/pre-exposure viable counts).

With respect to MIC stability, no experiments involving repeated or evolutionary exposures were performed. Under the defined strain, medium, and growth-condition combination used in this study, MIC values were reproducible across independent experiments. If needed, MIC values for shorter time points are directly reflected in the growth and kill kinetics presented in Figure 1.

If additional clarification regarding the time-kill assay is required, we would be happy to provide further detail. Our aim was to conduct the "growth" and "kill" experiments in as similar conditions as possible. Essentially, either OD for growth experiments or CFU/ml for kill experiments was measured from similar exposures. Unfortunately, both cannot be measured simultaneously from the same plate.

Toxicity of Cu and Ag depends strongly on ion speciation and chemical state. Without measurements or controls for free ion activity or complexation, reproducibility and cross-study comparability are uncertain.

> We agree that the toxicity of metal-based antimicrobials is strongly influenced by ion speciation and the chemical environment. As discussed in Supplementary Note 1 (section "*Exposure medium selection*"), metal speciation in biological systems is highly dynamic and depends on both abiotic factors (*e.g.,* medium composition, pH, chloride content) and biotic components (*e.g.,* biomolecules and dead cell matter), which continuously modulate metal bioavailability during exposure.
>
> In this study, a minimally complexing, MOPS-buffered defined medium was selected to reduce pH-driven and medium-dependent biases while allowing direct comparison of copper and silver effects under similar conditions. While direct measurement of free ion activity can be informative in simplified chemical systems, reliance on free ion concentration alone represents an oversimplification in dynamic biological exposures, where metal complexes may remain bioavailable and contribute to toxicity via alternative uptake routes. *E.g* metal-amino-acid complexes can hitchhike amino acid transporters and gain an uptake route due to which disruption of amino acid transporters can decrease toxic metal uptake as in the case of copper and silver (Rosenberg et al., 2025) or selenium (Deutch et al., 2014) and cystine transport via YdjN.
>
> Our experimental design therefore prioritized internal consistency and comparability of growth and kill kinetics between copper and silver rather than absolute quantification of free ion activity. The limitations associated with speciation and medium dependence are explicitly acknowledged, and the chosen conditions reflect a compromise that is both experimentally tractable and biologically relevant within the scope of this Observation.

Growth and killing trends are primarily interpreted visually. Explicit statistical modeling of growth rates, lag times, killing rates, and effect sizes would strengthen the conclusions.

> Growth and killing dynamics were not interpreted solely based on visual inspection. Growth parameters, including lag phase duration, doubling time, and biomass yield, were quantitatively extracted from growth curves and statistically analyzed as described in Supplementary Note 2. These analyses are summarized in Supplementary Figures S1 and S2 and support the reported dose-dependent effects of copper and silver on growth kinetics.

Visual presentation of representative growth and kill curves in Figure 1 was chosen to convey the overall kinetic behavior underlying the observation, while detailed parameterization and statistical testing were provided as supplementary information.

With respect to kill kinetics, we agree that rate-based modeling can be informative. However, the rapid initial killing followed by regrowth observed under silver exposure results in U-shaped kill–growth dynamics, complicating rate estimation without resolving whether killing and regrowth occur sequentially or overlap temporally. Addressing this would require experimental designs that differ from those used to maintain comparability between growth and kill assays and was therefore beyond the scope of the present study.

Claims regarding antimicrobial testing practices and risk assessment would be stronger if supported by at least one additional strain or species.

We agree that extending the analysis to additional strains or species would strengthen generalizability. However, the present study was intentionally designed and submitted as an *Observation* to report a focused kinetic phenomenon rather than to provide broad strain- or species-level validation.

As discussed in Supplementary Note 1 (sections *"Strain and inoculum selection"* and *"Re-growth conditions")*, strain selection and growth conditions represent acknowledged limitations of the study. The objective here was to demonstrate that sub-MIC copper and silver exposures can produce fundamentally different growth and kill kinetics that are not captured by standard endpoint assays, even within a single, well-defined experimental system.

We view this observation as hypothesis-generating and as motivation for future strain- and context-specific investigations, rather than as a comprehensive assessment of antimicrobial risk across taxa.

The manuscript draws broad implications for antimicrobial product evaluation and resistance development that may not be fully supported by the relatively compact dataset presented. While the manuscript raises an important conceptual point and presents solid preliminary data, its current form may not meet the evidentiary and scope expectations for a full research article. Substantial revision-particularly expanding experimental breadth, strengthening mechanistic or evolutionary support, and tightening the discussion-would be required.

We agree that the dataset presented is compact and that the manuscript is not intended to meet the evidentiary breadth, mechanistic depth, or experimental scope expected of a full Research Article. The study was therefore intentionally submitted as an *Observation* to report a focused kinetic finding supported by a limited but well-

defined dataset, rather than to provide comprehensive mechanistic, evolutionary, or product-level validation.

Accordingly, the discussion of antimicrobial testing practices, tolerance, resistance, and risk assessment is framed to highlight plausible implications of the observed kinetic differences between copper and silver, rather than to draw definitive or generalizable conclusions. We have aimed to clearly delimit the scope of the study, acknowledge its limitations, and present the findings as hypothesis-generating. Within the expectations of the Observation format, we believe that the current data sufficiently supports the central conceptual point that reliance on static endpoint assays such as MIC can obscure biologically relevant sub-MIC toxicity dynamics.

Reviewer #2 (Comments for the Author):

In this manuscript Rosenberg, et al examine growth behavior of E. coli in the presence of sub inhibitory concentrations of silver and copper, two well-known metals with antimicrobial properties. Overall the manuscript was well written and provided an interesting data set, described and discussed well by the authors. Included below are a few comments that the authors may wish to address to further strengthen the manuscript and add clarity for readers.

1. Figure one C/D was more difficult to interpret the significant of the range "whiskers". Not sure if some spots they were overlapping, but I struggled with them particularly at the right side of the two plots.

We thank the reviewer for this comment. In Figure 1C and 1D, viable counts are presented as mean ± standard deviation from three biological replicates, with the primary aim of illustrating temporal changes in survival rather than statistically comparing dose-dependent effects at individual time points.

To improve clarity, we added a bar-graph representation of the same survival data as Supplementary Figure S5, which facilitates interpretation of variability across conditions. The relatively large variability observed for longer exposures or higher silver concentrations reflects inherent biological and technical variability associated with cultivation-based viable count measurements, particularly under conditions of substantial killing. The raw data underlying these figures are provided in the Supplementary Data file.

2. Remove e.g. in line 110 or restructure the sentence.

We have rephrased the indicated sentence to improve readability, as suggested.

On the other hand, silver resistance is a well-known challenge necessitating combined treatment strategies, e.g. in burn wound care (17, 18).

3. I think the conclusion line could use further argument/discussion for justification or should be more directly correlated with the data in this observation manuscript.

We have revised the concluding sentence to more explicitly link the broader implications to the kinetic observations presented in this study. The revised wording emphasizes that the conclusions are based on the demonstrated limitations of endpoint MIC assays in capturing transient killing and regrowth dynamics, rather than on generalized claims about resistance development (addition in bold):

In conclusion, our observations highlight new and known challenges in assessing antimicrobial characteristics by using wide-spread endpoint assays such as MIC ***that mask toxicity kinetics*** *to make decisions guiding the design and risk assessment of antimicrobial formulations in different fields of application.*

Aboubakr, H.A., Sharafeldin, T.A., Goyal, S.M., 2021. Stability of SARS-CoV-2 and other coronaviruses in the environment and on common touch surfaces and the influence of climatic conditions: A review. Transbound. Emerg. Dis. 68, 296–312. https://doi.org/10.1111/tbed.13707

Balaban, N.Q., Helaine, S., Lewis, K., Ackermann, M., Aldridge, B., Andersson, D.I., Brynildsen, M.P., Bumann, D., Camilli, A., Collins, J.J., Dehio, C., Fortune, S., Ghigo, J.-M., Hardt, W.-D., Harms, A., Heinemann, M., Hung, D.T., Jenal, U., Levin, B.R., Michiels, J., Storz, G., Tan, M.-W., Tenson, T., Van Melderen, L., Zinkernagel, A., 2019. Definitions and guidelines for research on antibiotic persistence. Nat. Rev. Microbiol. 17, 441–448. https://doi.org/10.1038/s41579-019-0196-3

Blanco Massani, M., Klumpp, J., Widmer, M., Speck, C., Nisple, M., Lehmann, R., Schuppler, M., 2018. Chromosomal Sil system contributes to silver resistance in E. coli ATCC 8739. BioMetals 31, 1101–1114. https://doi.org/10.1007/s10534-018-0143-1

Bourge, M., Fort, C., Soler, M., Satiat-Jeunemaître, B., Brown, S.C., 2015. A pulse-chase strategy combining click-EdU and photoconvertible fluorescent reporter: tracking Golgi protein dynamics during the cell cycle. New Phytol. 205, 938–950. https://doi.org/10.1111/nph.13069

Deutch, C.E., Spahija, I., Wagner, C.E., 2014. Susceptibility of *Escherichia coli* to the toxic L-proline analogue L-selenaproline is dependent on two L-cystine transport systems. J. Appl. Microbiol. 117, 1487–1499. https://doi.org/10.1111/jam.12623

Fanous, J., Claudi, B., Tripathi, V., Li, J., Goormaghtigh, F., Bumann, D., 2025. Limited impact of Salmonella stress and persisters on antibiotic clearance. Nature 639, 181–189. https://doi.org/10.1038/s41586-024-08506-6

Gu, L., Saran, R., Yan, W., Huang, P.-J.J., Wang, S., Lyu, M., Liu, J., 2018. Reselection Yielding a Smaller and More Active Silver-Specific DNAzyme. ACS Omega 3, 15174–15181. https://doi.org/10.1021/acsomega.8b02039

Hong, R., Kang, T.Y., Michels, C.A., Gadura, N., 2012. Membrane Lipid Peroxidation in Copper Alloy-Mediated Contact Killing of Escherichia coli. Appl. Environ. Microbiol. 78, 1776–1784. https://doi.org/10.1128/AEM.07068-11

Hossain, Z., Huq, F., 2002. Studies on the interaction between Ag+ and DNA. J. Inorg. Biochem. 91, 398–404. https://doi.org/10.1016/S0162-0134(02)00454-3

Hosseini, M., Chin, A.W.H., Behzadinasab, S., Poon, L.L.M., Ducker, W.A., 2021. Cupric Oxide Coating That Rapidly Reduces Infection by SARS-CoV-2 via Solids. ACS Appl. Mater. Interfaces 13, 5919–5928. https://doi.org/10.1021/acsami.0c19465

Hötzer, B., Ivanov, R., Altmeier, S., Kappl, R., Jung, G., 2011. Determination of Copper(II) Ion Concentration by Lifetime Measurements of Green Fluorescent Protein. J. Fluoresc. 21, 2143–2153. https://doi.org/10.1007/s10895-011-0916-1

Hsueh, Y.-H., Lin, K.-S., Ke, W.-J., Hsieh, C.-T., Chiang, C.-L., Tzou, D.-Y., Liu, S.-T., 2015. The Antimicrobial Properties of Silver Nanoparticles in Bacillus subtilis Are Mediated by Released Ag+ Ions. PLOS ONE 10, e0144306. https://doi.org/10.1371/journal.pone.0144306

Jensen, G.C., Janis, M.K., Nguyen, H.N., David, O.W., Zastrow, M.L., 2024. Fluorescent Protein-Based Sensors for Detecting Essential Metal Ions across the Tree of Life. ACS Sens. 9, 1622–1643. https://doi.org/10.1021/acssensors.3c02695

Krewing, M., Mönch, E., Bolten, A., Niesalla, H., 2024. Resistance or tolerance? Highlighting the need for precise terminology in the field of disinfection. J. Hosp. Infect. 150, 51–60. https://doi.org/10.1016/j.jhin.2024.05.006

Kuffel, A., Gray, A., Daeid, N.N., 2021. Impact of metal ions on PCR inhibition and RT-PCR efficiency. Int. J. Legal Med. 135, 63–72. https://doi.org/10.1007/s00414-020-02363-4

Manuel, C.S., Moore, M.D., Jaykus, L.A., 2015. Destruction of the Capsid and Genome of GII.4 Human Norovirus Occurs during Exposure to Metal Alloys Containing Copper. Appl. Environ. Microbiol. 81, 4940–4946. https://doi.org/10.1128/AEM.00388-15

Mathews, S., Kumar, R., Solioz, M., 2015. Copper Reduction and Contact Killing of Bacteria by Iron Surfaces. Appl. Environ. Microbiol. 81, 6399–6403. https://doi.org/10.1128/AEM.01725-15

Mohapatra, B., Mohapatra, S., Sharma, N., 2023. Biosynthesized Ag–ZnO nanohybrids exhibit strong antibacterial activity by inducing oxidative stress. Ceram. Int. 49, 20218–20233. https://doi.org/10.1016/j.ceramint.2023.03.146

Moreno, L.I., McCord, B.R., 2017. Understanding metal inhibition: The effect of copper (Cu 2+) on DNA containing samples. Forensic Chem. 4, 89–95. https://doi.org/10.1016/j.forc.2017.03.005

Moshkovich, L., Saphier, O., Popov, S., Shotland, Y., Silberstein, E., Saphier, M., 2021. Monovalent Copper and Silver Ions Block DNA Polymerase Chain Reaction. https://doi.org/10.21203/rs.3.rs-543661/v1

Priester, J.H., Singhal, A., Wu, B., Stucky, G.D., Holden, P.A., 2014. Integrated approach to evaluating the toxicity of novel cysteine-capped silver nanoparticles to Escherichia coli and Pseudomonas aeruginosa. The Analyst 139, 954–963. https://doi.org/10.1039/C3AN01648J

Ronneau, S., Hill, P.W., Helaine, S., 2021. Antibiotic persistence and tolerance: not just one and the same. Curr. Opin. Microbiol. 64, 76–81. https://doi.org/10.1016/j.mib.2021.09.017

Rosenberg, M., Park, S., Umerov, S., Ivask, A., 2025. Experimental evolution of *Escherichia coli* on semi-dry silver, copper, stainless steel, and glass surfaces. Microbiol. Spectr. 13, e02173-24. https://doi.org/10.1128/spectrum.02173-24

Warnes, S. L., Caves, V., Keevil, C.W., 2012. Mechanism of copper surface toxicity in *Escherichia coli* O157:H7 and *Salmonella* involves immediate membrane depolarization followed by slower rate of DNA destruction which differs from that observed for Gram-positive bacteria. Environ. Microbiol. 14, 1730–1743. https://doi.org/10.1111/j.1462-2920.2011.02677.x

Warnes, S.L., Green, S.M., Michels, H.T., Keevil, C.W., 2010. Biocidal Efficacy of Copper Alloys against Pathogenic Enterococci Involves Degradation of Genomic and Plasmid DNAs. Appl. Environ. Microbiol. 76, 5390–5401. https://doi.org/10.1128/AEM.03050-09

Warnes, Sarah L., Highmore, C.J., Keevil, C.W., 2012. Horizontal Transfer of Antibiotic Resistance Genes on Abiotic Touch Surfaces: Implications for Public Health. mBio 3, e00489-12. https://doi.org/10.1128/mBio.00489-12

Warnes, S.L., Keevil, C.W., 2011. Mechanism of Copper Surface Toxicity in Vancomycin-Resistant Enterococci following Wet or Dry Surface Contact. Appl. Environ. Microbiol. 77, 6049–6059. https://doi.org/10.1128/AEM.00597-11

Warnes, S.L., Summersgill, E.N., Keevil, C.W., 2015. Inactivation of Murine Norovirus on a Range of Copper Alloy Surfaces Is Accompanied by Loss of Capsid Integrity. Appl. Environ. Microbiol. 81, 1085–1091. https://doi.org/10.1128/AEM.03280-14

Weaver, L., Noyce, J.O., Michels, H.T., Keevil, C.W., 2010. Potential action of copper surfaces on meticillin-resistant Staphylococcus aureus: Action of copper surfaces on MRSA. J. Appl. Microbiol. 109, 2200–2205. https://doi.org/10.1111/j.1365-2672.2010.04852.x

Yamamoto, A., Tanaka, S., Ohishi, K., 2021. Quantitative Evaluation of Nucleic Acid Degradability of Copper Alloy Surfaces and Its Correlation to Antibacterial Activity. Antibiot. Basel Switz. 10, 1439. https://doi.org/10.3390/antibiotics10121439

Re: Spectrum03512-25R2 (Distinct sub-MIC kill kinetics of Cu and Ag in *Escherichia coli*)

Dear Dr. Merilin Rosenberg:

Your manuscript has been accepted, and I am forwarding it to the ASM production staff for publication. Your paper will first be checked to make sure all elements meet the technical requirements. ASM staff will contact you if anything needs to be revised before copyediting and production can begin. Otherwise, you will be notified when your proofs are ready to be viewed.

Data Availability: ASM policy requires that data be available to the public upon online posting of the article, so please verify all links to sequence records, if present, and make sure that each number retrieves the full record of the data. If a new accession number is not linked or a link is broken, provide production staff with the correct URL for the record. If the accession numbers for new data are not publicly accessible before the expected online posting of the article, publication may be delayed; please contact ASM production staff immediately with the expected release date.

Publication Fees: For information on publication fees and which article types have charges, please visit our website. We have partnered with Copyright Clearance Center (CCC) to collect author charges. If fees apply to your paper, you will receive a message from no-reply@copyright.com with further instructions. For questions related to paying charges through RightsLink, please contact CCC at ASM_Support@copyright.com or toll free at +1-877-622-5543. CCC makes every attempt to respond to all emails within 24 hours.

ASM Membership: Corresponding authors may join or renew ASM membership to obtain discounts on publication fees. Need to upgrade your membership level? Please contact Customer Service at Service@asmusa.org.

Embargo Policy: A press release may be issued as soon as the manuscript is posted on the Spectrum Latest Articles webpage. The corresponding author will receive an email with the subject line "ASM Journals Author Services Notification" when the article is available online.

The ASM Journals program strives for constant improvement in our submission and publication process. Please tell us how we can improve your experience by taking this quick Author Survey.

Thank you for submitting your paper to Spectrum.

Sincerely,
Mor Lurie-Weinberger
Editor
Microbiology Spectrum

Reviewer #3 (Comments for the Author):

All my concerns have been addressed.

Reviewer #4 (Comments for the Author):

The respected authors have provided convincing answers to my comments, and I believe that the observational study is acceptable.
